# Efficacy and Safety of Rituximab for New-Onset Generalized Myasthenia Gravis: The RINOMAX Randomized Clinical Trial

**DOI:** 10.1001/jamaneurol.2022.2887

**Published:** 2022-09-19

**Authors:** Fredrik Piehl, Ann Eriksson-Dufva, Anna Budzianowska, Amalia Feresiadou, William Hansson, Max Albert Hietala, Irene Håkansson, Rune Johansson, Daniel Jons, Ivan Kmezic, Christopher Lindberg, Jonas Lindh, Fredrik Lundin, Ingela Nygren, Anna Rostedt Punga, Rayomand Press, Kristin Samuelsson, Peter Sundström, Oskar Wickberg, Susanna Brauner, Thomas Frisell

**Affiliations:** 1 Department of Neurology, Karolinska University Hospital, Stockholm, Sweden; 2 Neuroimmunology Unit, Center for Molecular Medicine, Karolinska Institutet, Stockholm, Sweden; 3 Department of Clinical Neuroscience, Karolinska Institutet, Stockholm, Sweden; 4 Department of Neurology and Rehabilitation, Ryhov Regional Hospital, Jönköping, Sweden; 5 Department of Neurology, Uppsala University Hospital, Uppsala, Sweden; 6 Department of Medical Sciences, Section of Neurology, Uppsala University, Uppsala, Sweden; 7 Department of Clinical Science, Neurosciences, Umeå University, Umeå, Sweden; 8 Department of Neurology, Linköping University Hospital, Linköping, Sweden; 9 Department of Biomedical and Clinical Sciences, Linköping University, Linköping, Sweden; 10 Department of Neurology and Rehabilitation, Central Hospital Karlstad, Karlstad, Sweden; 11 Department of Neurology, Sahlgrenska University Hospital, Gothenburg, Sweden; 12 Sahlgrenska Academy, Institute of Neuroscience and Physiology, Department of Clinical Neuroscience, Gothenburg University, Gothenburg, Sweden; 13 Clinical Neurophysiology, Department of Medical Sciences, Uppsala University, Uppsala, Sweden; 14 Department of Neurophysiology, Uppsala University Hospital, Uppsala, Sweden; 15 Clinical Epidemiology Division, Department of Medicine Solna, Karolinska Institutet, Stockholm, Sweden

## Abstract

**Importance:**

Rituximab is a third-line option for refractory generalized myasthenia gravis (MG) based on empirical evidence, but its effect in new-onset disease is unknown.

**Objective:**

To investigate the efficacy and safety of rituximab compared with placebo as an add-on to standard of care for MG.

**Design, Setting, and Participants:**

This randomized, double-blind, placebo-controlled study took place throughout 48 weeks at 7 regional clinics in Sweden. Key inclusion criteria were age older than 18 years, onset of generalized symptoms within 12 months or less, and a Quantitative Myasthenia Gravis (QMG) score of 6 or more. Patients were screened from October 20, 2016, to March 2, 2020. Key exclusion criteria included pure ocular MG, suspected thymoma, previous thymectomy, and prior noncorticosteroid immunosuppressants or high doses of corticosteroids.

**Interventions:**

Participants were randomized 1:1 without stratification to a single intravenous infusion of 500 mg of rituximab or matching placebo.

**Main Outcomes and Measures:**

Minimal disease manifestations at 16 weeks defined as a QMG score of 4 or less with prednisolone, 10 mg or less daily, and no rescue treatment.

**Results:**

Of 87 potentially eligible patients, 25 were randomized to rituximab (mean [SD] age, 67.4 [13.4] years; 7 [28%] female) and 22 to placebo (mean [SD] age, 58 [18.6] years; 7 [32%] female). Compared with placebo, a greater proportion with rituximab met the primary end point; 71% (17 of 24) in the rituximab group vs 29% (6 of 21) in the placebo group (Fisher exact test *P* = .007; probability ratio, 2.48 [95% CI, 1.20-5.11]). Secondary end points, comparing changes in Myasthenia Gravis Activities of Daily Living and Myasthenia Gravis Quality of Life at 16 weeks with QMG at 24 weeks did not differ between groups with censoring for rescue treatment (per-protocol analysis) but were in favor of active treatment when rescue treatment was taken into account by worst rank imputation (post hoc analysis). Rescue treatments were also more frequent in the placebo arm (rituximab: 1 [4%]; placebo, 8 [36%]). One patient in the placebo arm had a myocardial infarction with cardiac arrest and 1 patient in the active arm experienced a fatal cardiac event.

**Conclusions and Relevance:**

A single dose of 500 mg of rituximab was associated with greater probability of minimal MG manifestations and reduced need of rescue medications compared with placebo. Further studies are needed to address long-term benefit-risk balance with this treatment.

**Trial Registration:**

ClinicalTrials.gov Identifier: NCT02950155

## Key Points

**Question:**

Is a single infusion with rituximab associated with greater probability of having minimal disease manifestations at 4 months in recent-onset generalized myasthenia gravis?

**Findings:**

In this randomized clinical trial of 47 individuals, the proportion of individuals with minimal disease manifestations with only low doses of corticosteroids and no need of rescue treatment at 4 months was 71% with rituximab and 29% with placebo, respectively, indicating a significant difference.

**Meaning:**

Treatment with rituximab can be considered early after onset of generalized myasthenia gravis to reduce the risk of disease worsening and/or need of additional therapies.

## Teaser

This randomized clinical trial investigates the efficacy and safety of rituximab compared vs placebo as an add-on with standard of care for myasthenia gravis.

## Introduction

Myasthenia gravis (MG) is a prototypical autoantibody-mediated neuroimmunological condition with a prevalence in Sweden of 24.8 per 100 000 individuals.^1,2^ Most patients with MG carry serum acetylcholine receptor (AChR+) antibodies and more rarely antibodies targeting muscle-specific kinase (MuSK+) or lipoprotein receptor–related protein 4, while a proportion lack antibodies to known antigenic targets (seronegative MG).^1^ While disease severity varies widely, it is well acknowledged that among those with generalized symptoms, many experience substantial morbidity and sometimes even life-threatening events.^3,4^

In current treatment guidelines, mainly based on empirical experience and consensus agreements, oral corticosteroids, with daily doses up to 60 to 100 mg of prednisolone, are first-line therapy.^5^ Given the known short- and long-term adverse reactions with steroids, it is common practice to taper doses with addition of oral steroid-sparing immunosuppressive agents such as azathioprine, ciclosporin, methotrexate, mycophenolate, or tacrolimus.^5^ Several of these oral immunosuppressants have undergone randomized clinical trials with varying outcomes,^6,7,8,9,10,11^ while also being associated with adverse reactions and a long latency period before becoming effective,^5,12,13^ which leaves a substantial subgroup of patients with refractory symptoms.^14,15^ Biological treatments are considered third-line options, except in MuSK+ MG.^5,16^ However, only eculizumab, a complement inhibitor, holds a formal approval for use in refractory nonthymomatous AChR+ generalized MG and is associated with increased risk of severe infections and very high treatment cost.^13,17^ Hence, the need for effective, tolerable, and affordable drugs for MG remains.

Rituximab is a chimeric anti-CD20 monoclonal approved for B-cell lymphoma, rheumatoid arthritis, and vasculitis, which eliminates immature, naive, and memory B cells but not plasma cells.^18^ Therefore, at least theoretically, rituximab started early after disease onset might impede the buildup of a disease-associated plasma cell pool that otherwise would escape targeting.^19^ Indeed, in an observational study, we found preliminary evidence of improved effectiveness of rituximab compared with standard of care in patients with recent onset of generalized disease.^20^

To corroborate these findings in a controlled setting, we conducted a multicenter, placebo-controlled double-blinded randomized trial, the Rituximab in Patients With New-Onset Generalized Myasthenia Gravis) (RINOMAX) trial.

## Methods

### Study Population

RINOMAX was conducted at 5 Swedish university clinics and 2 larger regional neurology clinics enrolling participants from regional community-based catchment areas. Patients were screened from October 20, 2016, to March 2, 2020. Eligible patients were 18 years or older with onset of generalized MG symptoms 12 months or less prior to inclusion, with no time limit for isolated ocular symptoms, had a Quantitative Myasthenia Gravis (QMG) score of 6 or more (measured ≥12 hours since last dose of acetylcholinesterase inhibitors [AChEIs]) and a Myasthenia Gravis Foundation of America (MGFA) classification of II to IV. The MG diagnosis had to be confirmed by at least 2 of the following: a positive AChR antibody test result, an abnormal electrophysiological test result (repetitive nerve stimulation and/or single fiber electromyography) consistent with MG, and/or a clinically significant response to an oral or intravenous AChEI test (per treating physician’s opinion). The study was approved by the ethical committee of the Region of Stockholm (registration number 2016/870-31) and the Swedish Medical Products Agency, with written informed consent obtained from each participant.

The following exclusion criteria were applied: MGFA classification of I or V at screening, prior thymectomy or suspected thymoma based on radiology findings, significant comorbidity, use of immunosuppressive therapy including pulsed high-dose corticosteroids, rituximab, azathioprine, ciclosporin, and mycophenolate for any condition for 12 months or less prior to inclusion. Neither treatment with prednisolone, 40 mg/d or less, for a maximum of 3 months nor intravenous immunoglobulins or plasma exchange within 12 months of screening were considered exclusion criteria. The study protocol includes full inclusion/exclusion criteria and is available in Supplement 1.

Eligible participants were randomly assigned 1:1 without stratification to receive an intravenous infusion of 500 mg of rituximab or matched placebo. Randomization and preparation of blinded study drug were performed by a central pharmacy, Apoteket Produktion & Laboratorier in Stockholm, Sweden, and was shipped to study centers in identical liquid containers to preserve masking. Patients, investigators, and all study personnel were blinded throughout the study duration.

### Procedures, Data Collection, and Outcomes

The study drug was administered as a single intravenous infusion at the baseline visit, with 1000 mg of paracetamol, 10 mg of cetirizine, and 50 mg of prednisone administered as premedication. Use of AChEI as a symptomatic treatment was unrestricted, but doses were recorded at each visit. During the first 8 weeks, intravenous immunoglobulins and/or plasma exchange were not considered rescue treatment and could therefore be administered as needed. The use of prednisolone, 40 mg/d or less, was allowed but with tapering to arrive at 10 mg/d by study week 8. From week 9, standard of care medication with prednisolone, 10 mg/d or less, was permitted and noncorticosteroid oral immunomodulatory drugs were allowed from 12 weeks. Higher doses of prednisolone and all other immune modulatory treatments, including rituximab, intravenous immunoglobulins, and plasma exchange, were considered rescue treatments and classification as nonresponder (primary end point) and censoring (per protocol secondary end points), respectively.

Efficacy was evaluated with the QMG, Myasthenia Gravis Activity of Daily Life (MG-ADL), and Myasthenia Gravis Quality of Life (MG-QoL) scores. QMG was evaluated 12 hours or more after last intake of AChEI. Safety and tolerability were evaluated by documenting adverse events and serious adverse events. Assessments were performed at the screening and baseline visits and at 16, 24, 36, and 48 weeks after study drug infusion. Serum samples for AChR antibody analyses were collected at baseline and at 24 weeks and analyzed at the Department of Clinical Immunology, Karolinska University Hospital, using a standardized radioimmune assay. The study was independently monitored for compliance with good clinical practice. See Supplement 1 for the complete study flowchart.

The prespecified primary outcome was the proportion of patients with minimal disease manifestations defined as a QMG score of 4 or less and a daily dose of prednisolone of 10 mg/d or less at week 16, with no need of rescue treatment procedure(s) during study weeks 9 to 16. The 3 secondary outcomes were (1) change in QMG score between baseline and 24 weeks, (2) change in MG-ADL score between baseline and 16 weeks and, (3) change in MG-QoL score between baseline and 16 weeks. Tertiary outcomes included the primary end point evaluated at 24 weeks, proportion having received rescue treatment by week 24, change in QMG, MG-ADL, and MG-QoL scores at each study visit, hospitalization for worsened MG symptoms, and AChR antibody concentrations at week 24. Adverse events and serious adverse events were recorded to assess safety and tolerability. See Supplement 1 for all end points.

Certain amendments were made to the study protocol during the study, comprising prolonging the inclusion period, additions of study investigators, clarifying open-label treatment with rituximab at any time between baseline and 24 weeks as rescue therapy, and extending the time window for efficacy evaluation visits from ±7 to ±21 days.

### Statistical Analyses

We based the power calculation on literature data and our own earlier observational data (subsequently amplified and published)^20^ suggesting at most 40% meeting the primary end point with standard of care, while the corresponding proportion with active treatment would be 80%.^8^ Assuming no dropouts, 20 participants in each arm would give a power of 81% given a 2-sided test and a significance level of 95% (α = 5%), but to compensate for possible imbalances in randomization and a low rate of dropouts, our recruitment target was set at 45 participants. Descriptive statistics of baseline characteristics were tabulated by treatment arm and the magnitude of group imbalances were reported as standardized mean differences.

The primary end point (difference in proportions) was analyzed on an intention-to-treat basis with Fisher exact test and α = 0.05. The ratio of proportions was estimated in log-binomial regression with robust standard errors. The 3 secondary end points, defined as change in QMG, MG-ADL, and MG-QoL scores between baseline at specified evaluation visits, were independently analyzed with Mann-Whitney *U* tests among those with no rescue treatment before evaluation (per-protocol analysis) using a Bonferroni-adjusted α = 0.0167 to correct for multiplicity. Per the analysis plan, the mean difference between groups was also displayed with linear regression and 95% confidence limits estimated using robust standard errors and no multiplicity correction. All tertiary outcome measurements were exploratory. Binary tertiary end points were analyzed using Fisher exact test and continuous tertiary end points analyzed using the Mann-Whitney *U* test.

Certain exploratory analyses were done post hoc. These comprised (1) proportions with QMG minimal manifestations (≤4) without rescue therapy at each study visit, with differences tested with Fisher exact test; (2) time to rescue treatment displayed as Kaplan-Meier curves with difference tested in log-rank test and Cox regression of time since baseline; (3) intention-to-treat analysis for secondary end points using worst rank imputation for those receiving rescue therapy; and (4) correction for random imbalances between arms with exclusion of participants younger than 40 years and with certain comorbidity (fatal cardiac event and amyotrophic lateral sclerosis). All statistical analysis were done in SAS version 9.4 (SAS Institute).

## Results

**Figure 1.**
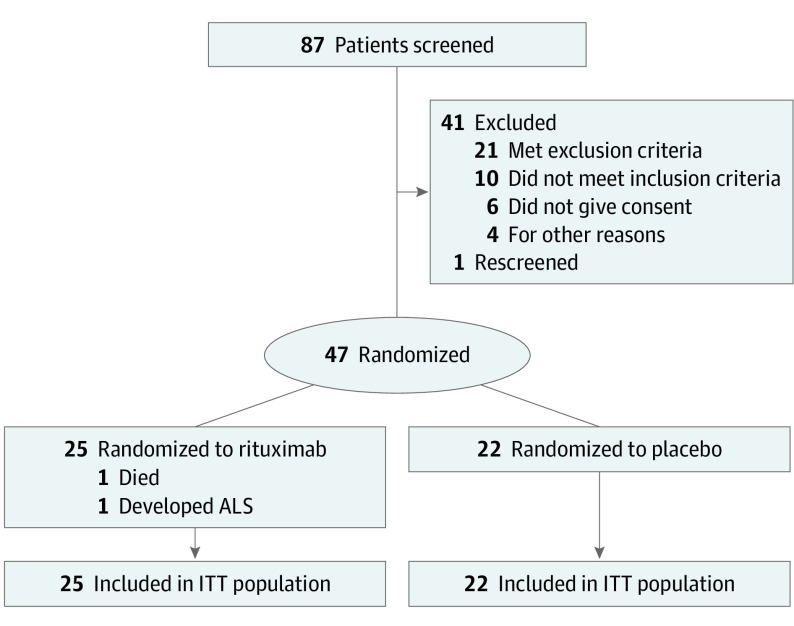
Study Flowchart ALS indicates amyotrophic lateral sclerosis; ITT, intention-to-treat.

A total of 87 potentially eligible patients were screened, of which 47 were enrolled (Figure 1). Overall, 25 individuals were randomized to rituximab (mean [SD] age, 67.4 [13.4] years; 7 [28%] female) and 22 to placebo (mean [SD] age, 58 [18.6] years; 7 [32%] female). Randomization was done without stratification and there were some resulting imbalances. Thus, the placebo group was younger, had higher AChR titers, and a higher proportion classified as MGFA III, whereas a higher proportion were taking oral prednisolone at baseline in the rituximab group (Table 1). The use of intravenous immunoglobulins prior to baseline was similar between arms; placebo: 8 of 22 (36%) and rituximab: 8 of 25 (32%), of which 1 had undergone plasma exchange. The proportions that received such treatments between baseline and end of week 8 were 55% with placebo (n = 22; 11 receiving intravenous immunoglobulins and 1 plasma exchange) and 28% with rituximab (n = 25; 7 receiving intravenous immunoglobulins). Prednisolone doses were increased from week 1 to 4 among 45% (n = 22) with placebo and 20% (n = 25) with rituximab, while the corresponding proportions from week 4 to 8 were 18% (n = 21) and 0%, respectively. All but 2 patients (who were also MuSK−) were AChR+, of which 8 had early-onset MG (age <50 years and AChR+). One participant in the placebo arm, presumed to have early-onset MG, had thymectomy at week 34 and was diagnosed with a thymoma.

**Table 1.** Baseline Demographics and Clinical Characteristics

Characteristic	No. (%)	
	Rituximab (n = 25)	Placebo (n = 22)
Age at inclusion, mean (SD), y	67.4 (13.4)	58 (18.6)
Female	7 (28.0)	7 (31.8)
Male	18 (72.0)	15 (68.2)
BMI, mean (SD)	27.5 (3.7)	27.6 (5.7)
Subtype		
EOMG	2 (8.0)	6 (27.3)
LOMG	23 (92.0)	15 (68.2)
Thymoma^a^	0	1 (4.5)
AChR+	23 (92.0)	22 (100.0)
AChR titer, nmol/L	25.1 (18.7)	70.7 (117.3)
Time since onset of generalized myasthenia gravis, mean (SD), d	132.4 (91.5)	143.0 (93.3)
Ocular symptoms prior to generalization	4 (16.0)	2 (9.1)
QMG score at baseline, mean (SD)	9.4 (4.5)	9.3 (4.2)
MG-ADL score at baseline, mean (SD)	5.1 (3.2)	4.5 (2.7)
MG-QoL score at baseline, mean (SD)	20.1 (11.0)	22.2 (12.8)
MGFA class at baseline^b^		
2a	2 (11.8)	3 (17.6)
2b	7 (41.2)	3 (17.6)
3a	1 (5.9)	1 (5.9)
3b	7 (41.2)	10 (58.8)
Prednisolone at baseline	16 (64.0)	12 (54.5)
Prednisolone dose, mean (SD), mg/d	22.5 (10.8)	20.8 (9.0)
Previous immunoglobulins	8 (32.0)	8 (36.4)
Previous plasmapheresis	1 (4.0)	0 (0.0)

Abbreviations: AChR+, seropositivity for acetylcholine receptor antibodies; BMI, body mass index (calculated as weight in kilograms divided by height in meters squared); EOMG, early-onset myasthenia gravis; LOMG, late-onset myasthenia gravis; MG-ADL, Myasthenia Gravis Activities of Daily Living; MG-QoL, Myasthenia Gravis Quality of Life; MGFA, Myasthenia Gravis Foundation of America; QMG, Quantitative Myasthenia Gravis.

^a^ Patient was initially diagnosed as having EOMG and was diagnosed with thymoma at study week 31.

^b^ Data available for 17 individuals in each arm.

**Figure 2.**
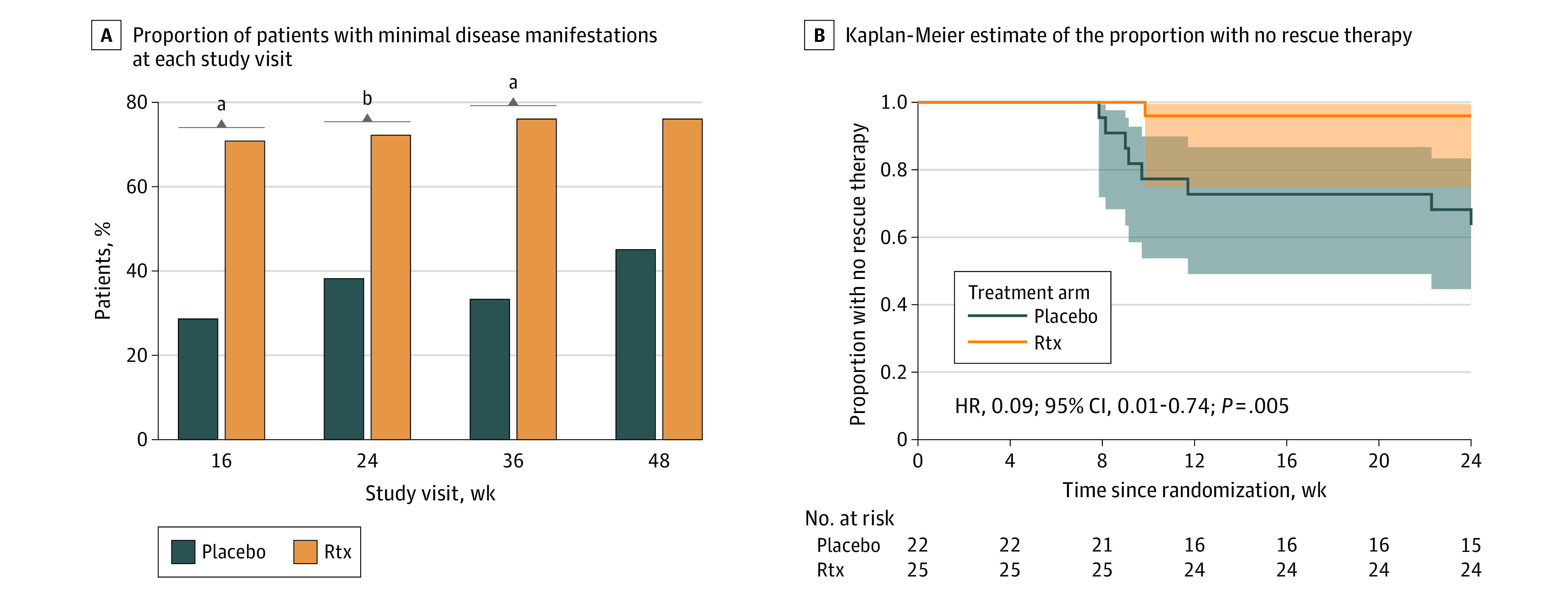
Proportion With Minimal Disease Manifestation and No Rescue Treatment Over Time A, Proportion with minimal disease manifestations at each study visit defined as Quantitative Myasthenia Gravis score ≤4 and a dose of prednisolone, ≤10 mg, without protocol-defined rescue treatment. Proportions at week 16 was the primary end point of the study, with other time points analyzed as tertiary end points. B, Kaplan-Meier estimates of the proportion with no rescue therapy and number of patients remaining at risk by week since randomization, for each treatment group. Shaded areas are 95% pointwise CIs. This was a post hoc analysis. HR indicates hazard ratio; Rtx, rituximab. ^a^*P* = .007. ^b^*P* = .04.

The primary outcome was met by 17 of 24 (71%) and 6 of 21 individuals (29%) in the rituximab and placebo arms, respectively, corresponding to a probability ratio (PR) of 2.48 (95% CI, 1.20-5.11; Fisher exact test *P* value = .007; Figure 2A and Table 2). The proportions fulfilling the same criteria at study week 24 (tertiary end point) and weeks 36 and 48 (post hoc analyses) similarly favored active treatment arm over placebo (Figure 2A).

**Table 2.** End Points and Sensitivity Analyses

Factor	No. (%)		*P* value^a^	Probability ratio or mean difference (95% CI)^a^
	Rituximab (n = 25)	Placebo (n = 22)		
Primary end point				
Minimal disease manifestations at week 16^b^	17 (71)	6 (29)	.007	2.48 (1.20 to 5.11)^c^
Secondary end points				
Change in QMG score, week 24^d^	−6.9 (5.6)	−5.8 (4.6)	.79	−1.1 (−4.4 to 2.1)
Change in MG-ADL score, week 16^e^	−1.7 (2.5)	−0.5 (3.6)	.34	−1.2 (−3.3 to 0.8)
Change in MG-QoL score, week 16^e^	−9.2 (9.2)	−7.0 (9.3)	.47	−2.2 (−8.2 to 3.8)
Tertiary end points				
Minimal disease manifestations and no rescue therapy at week 24^f^	18 (72)	8 (38)	.036	1.89 (1.04 to 3.44)^c^
Antibody titer, nmol/L	12.5 (21.0)	77.1 (159.0)	.16	−64.5 (−134.8 to 5.7)^c^
Hospitalization due to MG exacerbation	0	3	NA	NA
Patients receiving any rescue therapy before 24 wk	1 (4)	8 (36)	.008	0.11 (0.01 to 0.81)^c^
High-dose corticosteroids	1 (4)	5 (23)	NA	NA
Plasmapheresis	0	1 (5)	NA	NA
IVIG	0	6 (27)	NA	NA
Biologics (rituximab or tocilizumab)	0	5 (23)	NA	NA
Post hoc analyses (worst rank imputation)				
Change in QMG score, week 24^b^	−6.5 (5.9)	−2.0 (6.0)	.04	−4.4 (−7.8 to −1.0)
Change in MG-ADL score, week 16^g^	−1.3 (3.2)	2.0 (5.0)	.03	−3.2 (−5.6 to −0.8)
Change in MG-QoL score, week 16	−8.4 (10.2)	−2.1 (11.1)	.06	−6.8 (−12.4 to −0.1)

Abbreviations: IVIG, intravenous immunoglobulin; MG, myasthenia gravis; MG-ADL, Myasthenia Gravis Activities of Daily Living; MG-QoL, Myasthenia Gravis Quality of Life; NA, not applicable; PR, probability ratio estimated with log-binomial regression; QMG, Quantitative Myasthenia Gravis.

^a^ *P* value is Fisher exact test for binary end points and Kruskal-Wallis test for continuous variables. 95% CIs were estimated with robust (Huber-White) standard errors.

^b^ Missing data: rituximab, 1; placebo, 1.

^c^ PR (95% CI) is reported.

^d^ Censured patients, rituximab = 2, placebo = 9.

^e^ Censured patients, rituximab = 3, placebo = 7.

^f^ Missing data, rituximab = 0, placebo = 1.

^g^ Missing data, rituximab = 2, placebo = 1.

As sensitivity analyses assessing the impact of baseline differences in patient characteristics, we fitted 2 logistic regression models, crude and adjusted for age (cubic polynomial), early-onset disease, and MGFA classification. The crude odds ratio was 6.07 (95% CI, 1.67-22.1), and significance remained after adjustment (odds ratio, 4.63 [95% CI, 1.08-19.8]). We also ran a post hoc analysis excluding patients younger than 40 years and with comorbidity but with virtually unchanged results. In addition, prednisolone doses at baseline did not seem to predict the primary end point (data not shown).

The predefined secondary end points (with censoring for rescue treatment) did not differ between arms, but there was a disproportional loss of participants in the placebo arm: 3 and 7 at 16 weeks and 2 and 9 patients at week 24, in the rituximab and placebo arms, respectively (Table 2; eFigure 1 in Supplement 2). In a post hoc analysis using worst rank imputations for those receiving rescue treatment, change in QMG score between baseline and week 24 and change in MG-ADL score at week 16 favored rituximab over placebo, with a trend also for MG-QoL score change at week 16 (Table 2; eFigure 2 in Supplement 2). Analyzed as a tertiary end point, fewer patients in the rituximab group required rescue treatment compared with placebo (1 of 25 [4%] in the rituximab group vs 8 of 22 [36%] in the placebo group; PR, 0.11 [95% CI, 0.01-0.81]; *P* = .008). When displayed as a Kaplan-Meier curve (post hoc), rescue events were seen to occur more frequently in the placebo arm from week 9 until week 24 (Figure 2B). As mentioned previously, oral prednisolone doses at baseline tended to be higher in the rituximab arm; however, this relation shifted in the run-in period (week 1 to 8) (eTable in Supplement 2). Furthermore, 3 patients in the placebo group experienced MG exacerbations that required hospitalization (1 requiring invasive ventilation) compared with none in the rituximab group (Table 2). In contrast, there were no significant differences in AChR antibody titers between rituximab and placebo assessed at week 24 (predefined analysis; Table 2). However, in post hoc analyses, comparing changes in autoantibody titers from baseline to week 24, we observed a trend for reduction in the rituximab group not evident with placebo (eFigure 3 in Supplement 2).

The number of adverse events was greater in the rituximab group compared with placebo (81 vs 44), with 6 vs 4 severe adverse events in the rituximab and placebo arms, respectively (Table 3). One patient with MG with preexisting, but evaluated as stable, ischemic heart disease experienced a fatal cardiac event 4 weeks after baseline. Two patients in the placebo arm experienced life-threatening events but recovered without sequalae (1 with myocardial infarction with cardiac arrest in context of an MG exacerbation, and 1 with bacterial septicemia during an MG crisis). Mild infusion reactions were reported for 3 and 1 individuals in the rituximab and placebo arms, respectively.

**Table 3.** Adverse Events

Factor	No. (%)	
	Rituximab (n = 25)	Placebo (n = 22)
Adverse events	81	44
Patients with ≥1 event	21 (84)	18 (82)
Infusion reactions^a^	3 (12)	1 (5)
Severe adverse events	6 (24)	4 (18)
Patients experiencing ≥1 severe adverse event	5 (20)	3 (17)
Fatal severe adverse event	1 (4)	0
Life threatening	0	2 (9)
Hospitalization not due to exacerbation	4 (16)	2 (9)
Most common adverse events (≥10% in either group)		
Upper respiratory tract infections	7 (28)	8 (36)
Bacterial infections requiring systemic antibiotics	3 (14)	2 (9)
Musculoskeletal pain	8 (32)	5 (23)
Diarrhea	6 (24)	2 (9)
Nausea	4 (16)	1 (5)
Rash	3 (14)	3 (12)

^a^ All infusion reactions were mild, ie, grade 1 or 2 according to the Common Terminology Criteria for Adverse Events version 5.0.

## Discussion

Allocation to a low-dose rituximab protocol resulted in a higher proportion of patients with minimal disease manifestations, despite low corticosteroid doses and no use of rescue therapies, at 16 weeks compared with placebo. While we did not find statistically significant differences in the prespecified secondary outcomes, it is evident that censoring for rescue treatment affected the power for these analyses, with a disproportionate impact on the placebo arm. Rescue treatment was more frequently used in the placebo arm, where also all hospitalizations due to MG exacerbations, including a myasthenic crisis requiring mechanical ventilation, occurred. Accordingly, in post hoc analyses, taking rescue treatment into account by worst rank imputation, improvements in QMG and MG-ADL scores were greater with active treatment, with a trend also for a difference in improvement in MG-QoL scores.

A number of mainly retrospective observational studies have indicated a potential benefit of rituximab in generalized MG, in particular among individuals with MuSK+.^16,19,21^ In most cases this concerned refractory MG, ie, individuals with longer disease duration with previous or ongoing exposure to other immunosuppressive agents, as also reflected by current treatment guidelines.^5^ However, so far, evidence obtained in a controlled setting has been scarce. Recently the BeatMG placebo-controlled trial with rituximab was published.^22^ In this study, 52 participants with AChR+-generalized MG and ongoing corticosteroid treatment (prednisone, ≥15 mg/d) without limitation on disease duration were randomized to receive an infusion of 375 mg of rituximab per m^2^ on 4 consecutive weeks, or matching placebo, with follow-up throughout 52 weeks. The primary outcome of BeatMG was the steroid-sparing effect without exacerbation of MG, which was achieved by 60% and 56% in the rituximab and placebo arms, respectively. Study populations and procedures differed substantially between BeatMG and the present study, which likely explains the opposing findings. For example, there was no disease severity requirement for inclusion in the Beat-MG trial and there may exist a theoretical advantage of using rituximab earlier in the treatment algorithm based on immunological features of anti-CD20 monoclonals.^18,19^ This is in line with our previous observational study, where we observed a faster and more complete clinical response to rituximab in participants with recent onset of generalized MG compared with those with longer disease duration.^20^ This study applied a low-dose rituximab protocol frequently used for multiple sclerosis in Sweden. In a previous study, the rate of noninfusion-related adverse events tended to be less frequent with a single 500-mg infusion compared with 1000 mg, while levels of B cells at reinfusion did not differ.^23^ However, since a proportion of patients will display repopulation of B cells at reinfusion at 6 months and we only recorded a trend for lowered AChR antibody levels in the active treatment arm, also other mechanisms must be considered, such as elimination of CD20+ T and antigen-specific memory B cells, antigen presentation and modulation of cytokine production by B cells.^18^

The general short- to medium-term safety of rituximab was in line with previous literature. At the group level, no new safety signals emerged with the use of rituximab in new-onset generalized MG compared with its use in other indications, which includes higher risk of severe infections.^24^ One study participant with preexisting ischemic heart disease randomized to rituximab died of myocardial infarction with cardiac arrest. Myocardial infarction is reported as an adverse event of rituximab, but interestingly rituximab as a therapeutic intervention in acute myocardial infarction appeared safe in the setting of a clinical trial.^25^ In addition, a case of acute myocardial infarction with cardiac arrest occurred also in the placebo arm, here in the context of MG exacerbation. Another study participant with active treatment was diagnosed with amyotrophic lateral sclerosis shortly after 24 weeks of observation. This concerned a woman in her mid to late 50s with bulbar onset, where initial neurophysiology showed disturbed single-fiber electromyography but no signs of motorneuron disease. She was seronegative for AChR and MuSK antibodies but had an initial transient response to AChEI and immunoglobulins and therefore fulfilled inclusion criteria. During the observation period, she developed an asymmetric limb weakness, where a renewed diagnostic workup showed distinct neurophysiological signs of motorneuron disease and highly elevated cerebrospinal fluid neurofilament light levels. Indeed, concurrent MG may occur in a small proportion of patients with onset of motorneuron disease.^26^

### Limitations

This study has limitations. This included an imbalance in some of the baseline characteristics including a lower age, higher AChR antibody titers, a lower proportion treated with prednisolone, and a greater proportion with MGFA class III disease in the placebo group. A stratification procedure may have prevented some of the imbalance; however, use of stratification is generally discouraged given that reliable predictive factors for treatment response are lacking. In addition, we ran sensitivity analyses adjusting for baseline characteristics with virtually unchanged results. Further, 2 participants in the rituximab arm experienced comorbidity that effectively excluded the possibility to evaluate a treatment effect. A post hoc analysis excluding patients younger than 40 years and the 2 participants with severe comorbidity rendered very similar results. It should also be noted that late-onset MG typically has more severe disease with less chance of spontaneous remissions compared with early onset MG.^27,28^ It is also evident that the censoring of participants who received rescue treatment severely impacted the power to detect a difference in the secondary end points. Furthermore, the inclusion criteria stipulated a minimum QMG score of 6, ie, moderate severity, which meant that some participants may have displayed an adequate response to limited doses of corticosteroids or immunoglobulins only. On the other hand, this decision was balanced by the intention to treat as early as possible in the disease process and being aware that MG exacerbation may well occur later on.^20^ It is also important to acknowledge that robust predictive markers of disease severity early in the disease course are scarce and that, from a study perspective, the inclusion of patients with MG with milder disease would diminish any treatment effect of rituximab. Finally, further studies are needed to determine if the positive treatment effects recorded here would be further improved with higher initial doses of rituximab.

## Conclusions

In conclusion, we here observed that a single infusion of 500 mg of rituximab increased probabilities to attain minimal disease manifestations despite low doses of corticosteroids in the short to medium term, which was also reflected as lower likelihood of requiring rescue treatment or hospitalization for MG exacerbation. Further studies are needed to shed light on long-term benefit-risk balance with rituximab in generalized MG as well as to define predictive markers for disease severity early in the disease course.

## Notes

Supplement 1. Trial protocol

Supplement 2. eFigure 1. Change in disease activity score from randomization, with censoring at time of rescue treatment eFigure 2. Change in disease activity score from randomization eFigure 3. Acetylcholine receptor antibody concentrations, change from baseline to week eTable. Stratification for daily Prednisolone doses

Supplement 3. Data sharing statement
