# Supplementary material for: Efficacy and Safety of Rituximab for New-Onset Generalized Myasthenia Gravis: The RINOMAX Randomized Clinical Trial
Source: JAMA Neurol. 2022 Sep 19;79(11):1105–12. doi: 10.1001/jamaneurol.2022.2887 (PMC9486640; doi:10.1001/jamaneurol.2022.2887)
Supplement: Supplement 1. — Trial protocol [file jamaneurol-e222887-s001.pdf]

The RINOMAX Study

A RANDOMIZED, DOUBLE-BLIND, PLACEBO-CONTROLLED MULTICENTER STUDY EVALUATING THE SAFETY AND EFFICACY OF RITUXIMAB (Mabthera®) IN PATIENTS WITH NEW ONSET GENERALIZED MYASTHENIA GRAVIS.

Product:	Mabthera®
Substance:	Rituximab
EudraCT-number:	2015-005749-30
Sponsor:	Karolinska Institutet<br>Department of Clinical Neuroscience<br>171 77 Stockholm
Sponsor's representative and coordinating investigator:	Fredrik Piehl<br>Neuroimmunology Unit. Dept Clinical Neuroscience.<br>Karolinska Institutet<br>CMM L8:4 Karolinska University Hospital (Solna)<br>S171 76 Stockholm

Table of Contents

1 SUMMARY 5
2 ABBREVIATIONS 8
3 ADMINISTRATIVE INFORMATION 9
3.1 Sponsor and coordinating investigator 9
3.2 Study centers and participating investigators 9
3.3 Neurophysiologist 11
3.4 Biostatistician 11
3.5 Monitoring 11
4 SIGNATURES 12
5 BACKGROUND 13
5.1 Backgrund and rationale 13
6 STUDY QUESTIONS 14
6.1 Primary question 14
6.2 Secondary questions 14
6.3 Tertiary and explorative questions 14
7 ENDPOINTS 14
7.1 Primary endpoint 14
7.2 Secondary endpoints 14
7.3 Tertiary and explorative endpoints 14
8 DESIGN 15
8.1 Study design 15
8.2 Screening period 15
8.3 Randomization 15
8.4 Treatment, baseline (week 0) 16
8.5 Evaluation, observation period (24 weeks) 17
8.6 Follow up period (weeks 25-48) 17
8.7 Study end 18
9 STUDY PARTICIPANTS 19
9.1 Inclusion criteria 19
9.2 Exclusion criteria 19
9.3 Criteria for discontinuing participation 20
9.4 Screening log 20
9.5 Study personell 20
10 STUDY DRUG 20
10.1 Study drug 20
10.2 Description of study drug 20
10.3 Placebo 21
10.4 Packaging, labeling and handling of study drug 21
10.5 Randomization 21
10.6 Blinding and emergency code breaking 21
10.7 Permitted / Not permitted treatments in different study phases 21

10.8 Clinical worsening, rescue treatments 22
10.9 Study drug documentation, accountability 23
10.10 Treatment after study end 23

11 ASSESSMENT OF SAFETY AND EFFICACY 23

11.1 Blood samples 23
11.2 Quantitative Myasthenia Gravis scale, QMG 23
11.3 Myasthenia gravis activities of daily life scale, MG-ADL 23
11.4 EuroQol 5-dimensions quality of life, EQ5D 23
11.5 Quality of life in myasthenia gravis, MG-QOL15 24
11.6 Neurophysiology 24
11.7 Adverse Event, AE and Serious Adverse Event, SAE. 24

12 MANAGMENT OF ADVERSE EVENTS 25

12.1 Definitions 25
12.2 Assessement of adverse events 25
12.3 Methods for detection of adverse events 25
12.4 Reporting of adverse events 26
12.5 Follow up of adverse events 26
12.6 Annnual safety reporting (Development Safety Update Report, DSUR) 26

13 STATISTICS, DATA HANDLING AND ARCHIVING 27

13.1 Deciding on number of study subjects 27
13.2 Statistical analyses 27
13.3 Data handling and Case Report Forms, CRF 27
13.4 Data processing 28
13.5 Archiving 28

14 QUALITY CONTROL 28

14.1 Source data 28
14.2 Monitoring 28
14.3 Access to source data 28

15 ETHICS 28

15.1 Ethics review board and medical products agency 28
15.2 Ethical conditions for the study 28
15.3 Risk benefit assessement 29
15.4 Collection of informed consent 29

16 INSURANCE 30

17 FUNDING 30

18 PUBLICATION OF RESULTS 30

19 REFERENCES 31

20 ANNEX 1, FLOW CHART 32

21 ANNEX 2, MGFA CLASSIFICATION 33

22 ANNEX 3, STUDY DRUG INFUSION/OBSERVATION 34

23 ANNEX 4, QMG 36

24 ANNEX 5, MG-QOL15 38

38

25 ANNEX 6, MG-ADL 39
26 ANNEX 7, EQ5D 40

94 # 1 Summary

PROTOCOL IDENTITY AND STUDY OBJECTIVE	
EudraCT-number:	2015-005749-30
Protocol number	2015-00887
Protocol title:	A randomized, double-blind, placebo-controlled multicenter study evaluating the safety and efficacy of Rituximab (Mabthera®) in patients with new-onset generalized Myasthenia gravis (MG).
Study objective	To evaluate the safety and efficacy of rituximab (Mabthera) in the treatment of patients with recent onset MG.

STUDY DRUG	
Product:	Mabthera (Rituximab)
Pharmaceutical formula:	Concentrate for infusion, solution
Dose	500 mg
Mode of administration:	Intravenous

METODOLOGY	
Study design:	Randomized, double-blind, placebo-controlled multicenter study
Primary question:	Is rituximab more effective than placebo at achieving minimal clinical MG symptoms without the need for higher doses of oral corticosteroids or rescue treatments at 16 weeks post-treatment?
Primary endpoint:	Proportion of patients with QMG score ≤ 4 and a daily dose of Prednisolone of ≤ 10mg and no rescue treatment at 16 weeks after study start

STUDY POPULATION	
Study subjects:	Patients with new onset generalized myasthenia gravis (MG)
Study size:	N=47
Inclusion criteria	1. Patients with oculobulbar, bulbar or generalized MG ≥ 18 years of age and ≤ 12 months after onset of generalized symptoms or neurophysiological detection of generalized disease.<br>2. The diagnosis of MG should be made by the following tests:<br>Clinical neurological status with motor symptoms compatible with MG and ≥ 2 of the following:<br>a Positive serological test for anti-acetylcholine receptor antibodies (AChR), and/or<br>b. For MG typical findings on neuro-physiological testing of neuromuscular

	transmission by single fiber electromyography (SFEMG) and / or repetitive nerve stimulation (RNS),<br>**and/or**<br>c. Positive anticholinesterase test, e.g. edrophonium chloride test or improvement of MG symptoms with oral cholinesterase inhibitors at the discretion of the treating physician.<br>3. MGFA clinical classification class II to IV at screening.<br>4. Quantitative MG score ≥ 6 at screening<br>5. Women of childbearing potential must have a negative pregnancy test.<br>6. Patients must have given written informed consent.<br>7. Patients must be able and willing to follow all study procedures.
Exclusion criteria	Weakness affecting only ocular or periocular muscles (MGFA class I).<br>2. MG crisis at screening (MGFA class V)<br>3. Thymectomy already performed. To avoid difficulties in evaluating the effect of the study drug, thymectomy, in cases where it is deemed indicated, should be planned for the follow-up period, i.e. only after the first 24 weeks.<br>4. Strong suspicion of thymoma and where thymectomy according to the treating physician should be performed within 24 weeks.<br>5. Active cancer, unless adequately treated<br>6. Pregnancy or breast-feeding.<br>7. Any ongoing acute or chronic viral or systemic bacterial infection including HIV and latent hepatitis B, which is clinically significant in the opinion of the study physician, and which has not been treated with appropriate antibiotic / antiviral drugs.<br>8. Severe heart failure (New York Heart Association Class IV) or severe uncontrolled heart disease<br>9. Previous use of immunosuppressive drugs including rituximab, azathioprine, ciclosporin and MMF. The use of Prednisolone at a dose of ≤40mg / d within 3 months and IVIG and PLEX 12 months from the screening date does not constitute an exclusion criterion. Note that this does not apply to treatment with immunosuppressive drugs / corticosteroids (excluding rituximab) for indications other than MG, provided that> 12 months have elapsed since the end of treatment.<br>10. Hypersensitivity to the active substance, to murine proteins or to any of the other excipients in the study medicinal product<br>11. Participation in any other clinical drug study or exposure to any other study drug,

	study product or study procedures within 30 days before screening.<br>12. Any medical condition which, in the opinion of the study physician, may interfere with the patient's participation in the study, poses any additional risk to the patient, or which complicates the assessment of the patients.<br>13. Vaccination within 4 weeks before inclusion

---

TIDSPLAN

Study period:	Q4 2016 – Q1 2021
Recruitment period:	Q4 2016 – Q1 2020

2 Abbreviations

**Förkortning**	**Förklaring**
AE	Adverse Event
APL	Apotek Produktion & Laboratorier AB
CMM	Center for Molecular Medicine
DSUR	Development Safety Update Report
EQ5D	EuroQol 5-dimensions
GCP	Good Clinical Practice
ICH	International Conference on Harmonisation
IV	Intravenous
IVIG	Intravenous immunoglobulines
KI	Karolinska Institutet
MG	Myasthenia gravis
MGFA	Myasthenia Gravis Foundation of America
MG-QOL 15	Quality of life - myasthenia gravis
MMF	Mycophenolate mofetil (Cellcept)
RNS	Repetitive nerve stimulation
SAE	Serious Adverse Event
SUSAR	Suspected Unexpected Serious Adverse Event
QMG	Quantitative Myasthenia Gravis scale

3 Administrative information

3.1 Sponsor and coordinating investigator

Sponsor:	Karolinska Institutet<br>Department of Clinical Neuroscience<br>171 77 Stockholm
Sponsor´s representative/<br>coordinating investigator	Fredrik Piehl, Neuroimmunology Unit. Dept Clinical Neuroscience<br>CMM L8:4 Karolinska University Hospital (Solna)<br>S171 76 Stockholm
Telephone:	+46 8 51779840 or +46-736718101
Fax:	+46 8 51773757
E-mail:	fredrik.piehl@ki.se

3.2 Participating study centers

3.2.1 Karolinska University Hospital

3.2.1.1 Karolinska University Hospital, Solna

Neurology Clinic
171 76 Stockholm

<u>Principal investigator:</u>

Fredrik Piehl — fredrik.piehl@ki.se

<u>Co-investigator:</u>

Albert Hietala — hietala@mac.com

3.2.1.2 Karolinska University Hospital, Huddinge

Neurology Clinic
141 86 Stockholm

<u>Principal investigator:</u>

Rayomand Press — Rayomand.press@karolinska.se

<u>Co-investigators:</u>

Ivan Kmezic — ivan.kmezic@gmail.com

Katharina Fink — katharina.fink@karolinska.se

Kristin Samuelsson — kristin.samuelsson@karolinska.se

3.2.2 Akademic hospital Uppsala

Neurology Clinic
Akademiska sjukhuset
751 85 Uppsala

<u>Principal investigato</u>

Ingela Nygren — ingela.nygren@akademiska.se

<u>Co-investigator</u>

Anna Rostedt Punga — annarostedtpunga@gmail.com

Amalia Feresiadou — feresiadouamalia@gmail.com

3.2.3 Central hospital Karlstad

Neurology Clinic
Centralsjukhuset
Rosenborgsgatan 2
651 85 Karlstad

<u>Principal investigator</u>
Rune Johansson Rune.Johansson@liv.se
<u>Co-investigator</u>
Oskar Wickberg oskar.wickberg@liv.se

3.2.4 University Hospital Linköping

Neurology Clinic
Universitetssjukhuset i Linköping
581 85 Linköping

<u>Principal investigator</u>
Fredrik Lundin fredrik.lundin@regionostergotland.se

<u>Co-investigator</u>
Irene Håkansson irene.hakansson@regionostergotland.se

3.2.5 Sahlgrenska University Hospital

Neurology Clinic
Sahlgrenska Universitetssjukhuset
413 46 Göteborg

<u>Principal investigator</u>
Daniel Jons daniel.jons@vgregion.se

<u>Co-investigator</u>
Christopher Lindberg christopher.lindberg@vgregion.se

3.2.6 Norrland´s University Hospital

Neurology Clinic
Norrlands Universitetssjukhus
901 85 Umeå

<u>Principal investigator</u>
Peter Sundström peter.sundstrom@umu.se

<u>Co-investigator</u>
Mattias Vågberg mattias.vagberg@umu.se

3.2.7 Örebro University Hospital

Neurology Clinic
Universitetssjukhuset Örebro
701 85 Örebro

<u>Principal investigator</u>
Martin Gunnarsson martin.gunnarsson@regionorebrolan.se

<u>Co-investigator</u>
Anna Wittrin anna.wittrin@regionorebrolan.se

3.2.8 Östersund´s Hospital

Östersunds sjukhus
Neurologmottagningen
831 83 Östersund

Principal investigato

Pierre De Flon pierre.deflon@regionjh.se

3.2.9 Ryhov´s Regional Hospital

Neurology Section
Department of Medicine
Länssjukhuset Ryhov
551 85 Jönköping

Principal investigato

Anna Budzianowska anna.budzianowska@rjl.se

Co-investigator

Jonas Lind jonas.lind@rlj.se

3.2.10 Skåne´s University Hospital

Neurology Clinic
Skånes Universitetssjukhus
Jan Waldenströms gata 19
222 41 Lund

Principal investigato

Andreea Ilianca andreea.ilianca@skane.se

Co-investigator

Fredrik Buchwald fredrik.buchwald@skane.se

3.3 Neurophysiologist

Anna Rostedt Punga annarostedtpunga@gmail.com
Neurophysiology Clinic
Akademiska sjukhuset
751 85 Uppsala

3.4 Biostatistician

Thomas Frisell thomas.frisell@ki.se
Department of Medicine
Karolinska Institutet
171 76 Stockholm

3.5 Monitoring

Karolinska Trial Alliance, kta@karolinska.se
KTA Support
Norra Stationsgatan 67
171 76 Stockholm

4 Signature page

Sponsor/
coordinating investigator

Fredrik Piehl
Karolinska Institutet
Department of Clinical Neuroscience
Stockholm

Signature   Date

Investigator
Name, title
Clinic
Hospital

5 Background

5.1 Background and rationale

Myasthenia gravis (MG) is a relatively rare autoimmune disease of the neuromuscular junction, caused by a defective neuromuscular transmission, which results from autoantibodies that bind to proteins involved in signaling at the neuromuscular synapse. MG is characterized by weakness and fatigue in skeletal muscles and occurs at all ages, but mainly among young adult women and in people of both sexes over 60 years. About 2,500 people in Sweden have the disease according to a recent epidemiological study[1].

The disease has a large variation in severity, where in more severe cases you need intensive care for a shorter or longer periods of time. Traditionally, MG is treated with purely symptom-relieving drugs and immunomodulatory / suppressive drugs. Pure symptom-relieving cholinesterase inhibitors can be sufficient in milder cases, but immunosuppressive agents must often be used to avoid persistent symptoms, which can be life-threatening as a result of failing respiratory functions.

MG is traditionally treated with high doses of corticosteroids (Prednisolone up to 60 mg / day) over a longer periods of time, which carries significant risks of side effects. Since the 1970s, therefore, oral immunosuppressive drugs have also been used as corticosteroid-sparing agents. This group includes azathioprine (Imurel), cyclosporine (Sandimmun) and mycophenolate (Cellcept). None of these drugs have the indication MG and the onset of effect is usually delayed.

In the largest randomized study to date in MG, in which 176 patients with persistent symptoms were randomized 1: 1 to mycophenolate or placebo, the active arm showed no significant improvement over placebo over 6 months on the primary endpoint; the proportion of patients with minimal symptoms and a daily dose of prednisolone ≤ 10 mg per day (44% in active arm versus 39% in placebo)[2]. In another study, 80 patients with mild to moderate generalized AChR-positive MG and a daily dose of 20 mg Prednisolone were randomized to MMF and placebo, respectively, and followed for 12 weeks with MMF. The primary efficacy parameter was change in the quantitative MG (QMG) scale[3], which did not differ significantly between the study arms ($p = 0.71$)[4]. These study results show that a significant proportion of MG patients continue to have generalized weakness involving limb weakness and bulbar symptoms (difficulty chewing, swallowing, speaking, and breathing) despite adequate dosing of immunosuppressive therapy. There is thus a great need to test newer treatment algorithms, which may include more effective biological drugs.

Several minor observational studies have shown that rituximab, an anti-CD20 monoclonal antibody that eliminates B cells, may have beneficial effects in treatment-refractory MG. There are currently a couple of randomized drug trials with rituximab using a haematological treatment protocol with repeated dosing of the drug for several weeks, with a total dose of over 2,000 mg. At Karolinska University Hospital, in recent years, more than 30 patients with MG, both refractory and new-onset patients, have been treated with rituximab, with a single infusion of 500 mg with good results and without serious side effects. In some cases, the treatment has been repeated after 6-12 months. An interesting observation is that the effect of the treatment seems to come much earlier than traditional immunosuppressive drugs, which leads to a reduced risk of hospitalizations and exposure to unnecessarily high cortisone doses. These experiences warrant further investigation of the efficacy and safety of rituximab in newly-diagnosed MG patients with moderate-to-severe disease and a low-dose protocol. The same dose (500 mg) is used by us for multiple sclerosis, where we currently have about 500 patients under treatment at Karolinska University Hospital.

The aim of the current study is to study the effect of rituximab compared to placebo in the treatment of newly debuted MG of at least moderate severity and with a risk of deterioration, i.e. subjects for whom prednisolone administration, repeated doses of IVIG or the addition of immunosuppressive medication may be indicated.

6 Study questions

6.1 Primary question

Is rituximab more effective than placebo at achieving minimal clinical MG symptoms without the need for higher doses of oral corticosteroids or rescue treatments at 16 weeks post-treatment?

6.2 Secondary questions

Is rituximab more effective than placebo at achieving improvement in standardized muscle fatigue testing at 24 weeks post-treatment?
Is rituximab more effective than placebo at achieving an improvement in the ability to perform activities of daily living at 16 weeks after treatment?

Is rituximab more effective than placebo at achieving an improvement in perceived quality of life at 16 weeks post-treatment?

6.3 Tertiary and explorative questions

Is rituximab more effective than placebo at achieving improvement in standardized muscular fatigue tests at 16, 36 and 48 weeks after treatment?

Is rituximab more effective than placebo at achieving differences in the ability to perform activities of daily living at 24, 36 and 48 weeks after treatment?

Is rituximab more effective than placebo at achieving an improvement in perceived quality of life at 24, 36 and 48 weeks after treatment?

Is rituximab more effective than placebo at achieving normalization of repetitive nerve stimulation at 24 weeks post-treatment?

To study the safety and effect of rituximab on the need for care in the treatment of MG

To study drug antibodies and immunological markers 6 months after treatment

7 Endpoints

7.1 Primary endpoint

Proportion of patients with QMG score ≤ 4 and a daily dose of Prednisolone of ≤ 10mg and no rescue treatment at 16 weeks after study start

7.2 Secondary endpoints

QMG scores at 24 weeks after treatment

MG-ADL scores at 16 weeks after treatment

MG-QOL scores at 16 weeks after treatment

7.3 Tertiary and explorative endpoints

Proportion of patients with QMG score ≤ 4 and a daily prednisolone dose ≤ 10mg and no rescue treatment at 24 weeks after treatment

QMG scores at 16, 36 and 48 weeks after treatment

MG-ADL scores at 24, 36 and 48 weeks after treatment

EQ5D scores at 16, 24, 36 and 48 weeks after treatment

MG-QOL scores at 24, 36 and 48 weeks after treatment

Hospital admissions

Rescue treatments

Adverse events

Autoantibody levels 24 weeks after treatment

Blood samples for exploratory immunological analyses at baseline, 16 and 24 weeks after treatment

8 Design

8.1 Study design

The study is a randomized, double-blind, parallel group, placebo-controlled multicenter study aimed at evaluating the safety and efficacy of rituximab (Mabthera) in the treatment of patients with new-onset MG. The study is expected to be carried out at 10 -15 centers in Sweden. In the study, approximately 50 individuals will be randomized 1: 1 to one of two treatment arms, (1) rituximab or (2) placebo infusion (NaCl).

Possible study participants are informed orally and in writing about the study during in- or outpatient care at the respective neurology clinic and can, after signed consent, be included in the study. *No study-related measures can be carried out before the consent is signed.*

8.2 Screening period

If the patient agrees to participate in the study, the following examinations are performed:

• Review of medical history and current medication

• Somatic status: including general condition (blood pressure, heart rate, body temperature), age, sex, height, weight and disease activity.

• Evaluation of muscle strength, QMG. If the patient has <6 in QMG, the evaluation must be repeated after at least 6 hours since last dose of cholinesterase inhibitors

Repetitive neurophysiology, RNS, for study subjects with a neurophysiology clinic in the hospital. See section 11.6, Neurophysiology

• Questionnaire regarding quality of life, EQ5D and MG-QoL15

• Ability to perform daily activities, MG-ADL.

• Blood tests: CBC incl. differential, Na, K, Creatinine, Asat, Alat, CRP, Glucose, hepatitis B screening, total IgG.

• Pregnancy test if the patient is a woman of childbearing age.

All women of childbearing potential are instructed to use effective contraception throughout the study after receiving the study drug. One of the following counts as an effective contraceptive:

* Combined hormonal contraceptives (estrogen and progestin) that inhibit ovulation

-oral, intravaginal, or transdermal

* Progesterone -hormonal contraceptives that inhibit ovulation

-oral, injectable, or implantable

* Spiral

* Bilateral fallopian tube occlusion

* Vasectomized partner

* Sexual abstinence, to refrain from heterosexual intercourse throughout the study.

8.3 Randomization

If all inclusion and exclusion criteria are met, the patient will be contacted to agree on a time for study drug administration. Apotek Produktion & Laboratorier, APL, is then contacted for randomization and ordering of study drug.

The study drug is delivered to the centers in ready-made infusion bags with refrigerated transport and temperature log by World Courier. The study drug is stored after delivery from APL in a refrigerator (+ 2-8 ◦C) until administration.

In addition to the study drug, study participants may receive other immunosuppressive treatment according to what is listed in the protocol under section 10.7, Permitted / non-permitted treatment at different times.

8.4 Study drug administration, baseline (V.0)

Study drug administration is carried out at a ward or outpatient clinic, depending on whether the patient is hospitalized or policlinic.

Inclusion and exclusion criteria are reconciled and QMG, MG-ADL, MG-QOL15, EQ5D are performed and somatic status is checked.

Note that QMG testing should be performed ≥12 hours after last dose of cholinesterase inhibitors.

To reduce the risk of allergic reactions, 1,000 mg paracetamol (Alvedon), 50 mg prednisone (Deltison) and 10 mg Cetirizine are administered at least 45 minutes before the infusion. Blood pressure, pulse and temp are checked before starting the infusion.

Important: Before starting the infusion, take a serum tube for exploratory immunological analysis.

The blood sample is sent to the sample management Karolinska University Hospital Solna, attach study-specific consultant referral.

Study drug is administered as an intravenous infusion (2 mg rituximab / ml, total 500 mg or placebo) with increasing infusion rate for a total of about 150 minutes by the nurse in charge of the infusion with the physician in charge of the infusion as support. See Appendix 3, Mabthera Infusion Monitoring.

Speed of Infusion

0-30 min	50ml/h
30-60	100ml/h
60-90 min	150ml/h
90 min -	200ml/h

In case of allergic reactions, the infusion is stopped temporarily and if necessary, 0.5 - 1 mg clemastine (Tavegyl) iv and / or 100 mg hydrocortisone (Solu-Cortef) can be administered intravenously. If the symptoms subside, the infusion can be restarted at the initial drip rate. In case of more severe reactions affecting systemic condition or with circulatory effects, the infusion should be stopped permanently.

The patient is monitored throughout the infusion and blood pressure, pulse and temperature are checked again after the end of the administration. The patient is then observed for another 60 minutes.

Important: before returning home, make sure the patient has:

- *Current contact information*
- *Informed to contact new / aggravated symptoms urgently, no later than 48 hours.*
- *Received instructions on medication with Prednisolone schedule if it has been deemed indicated*

The prednisolone schedule listed below may, if Prednisolone has not already been started on a purely clinical indication, be initiated immediately after the screening visit, or alternatively the day after administration of study drugs, if cortisone treatment is judged clinically indicated by the treating physician:

Starting dose 40 mg / day, with tapering of 5 mg / week until discontinuation.

For patients already on Prednisolone before screening, the pre-screening dose may be increased to 40 mg / day with subsequent tapering according to the above schedule.

To reduce the risk of study endpoints being affected by another MG-specific treatment, such treatment should be given after randomization in accordance with section 10.7, Permitted / non-permitted treatment at different times.

If the cortisone dose can not be reduced due to persistent or worsening clinical symptoms, you may first choose to increase the dose of Prednisolone and make a new plan for phasing out or, secondly, to give rescue treatment with IvIg or PLEX.

Immunosuppressive therapy (azathioprine, ciclosporin, MMF, methotrexate) can be started if indicated from 12 weeks after randomization. Pulse cures with cortisone should not be given after randomization. In cases where the study physician for medical reasons considers that the above treatments are insufficient or inappropriate, other treatment may also be considered, including rituximab. The patient will then be classified as a non-responder in the primary outcome measure and censored in the secondary outcome measures.

8.5 Evaluation, observation period (24 weeks)

After the single administration of study drug, the effect is followed up according to the following schedule:

• Telephone contact with the study nurse after 2 weeks (+/- 2 days): checking for side effects, verifying current medication and Prednisolone dose.

• Return visit after 4 weeks (+/- 2 days): checking for side effects, verifying current drugs and Prednisolone dose, QMG, MG-ADL, MG-QoL15, EQ5D and somatic status.

Note that QMG testing should be performed 12 hours after the last dose of cholinesterase inhibitors and preferably at the same time of day throughout all testing sessions.

• Telephone contact with a nurse after 8 weeks (+/- 4 days): checking for side effects, verifying current medication and Prednisolone dose.

• Telephone contact with a nurse after 12 weeks (+/- 4 days): checking for side effects, verifying current drugs and Prednisolone dose.

• Return visit after 16 weeks (+/- 21 days): checking for side effects, verifying current drugs and Prednisolone dose, QMG, MG-ADL, MG-QoL15, EQ5D and somatic status.

Note that QMG testing should be performed 12 hours after the last dose of cholinesterase inhibitors.

Important: a serum tube for exploratory immunological analyzes is taken and sent to the sample management Karolinska University Hospital Solna, attach study-specific consultant referral.

• Telephone contact with a nurse after 20 weeks (+/- 7 days): checking for side effects, verifying current drugs and Prednisolone dose.

• Return visit after 24 weeks (+/- 21 days): checking for side effects, verifying current drugs and Prednisolone dose, QMG, MG-ADL, MG-QoL15, EQ5D and somatic status.

Note that QMG testing should be performed 12 hours after the last ingestion of cholinesterase inhibitors.

Important: a serum tube for exploratory immunological analyzes is taken and sent to the sample management Karolinska University Hospital Solna, attach study-specific consultant referral.

At the centers that have a neurophysiology clinic at the hospital, a repetitive nerve stimulation will be performed in connection with the return visit or in close proximity to the visit, see section 11.6 Neurophysiology.

8.6 Follow up period (25-48 weeks)

After a 24-week evaluation phase, the study moves to an open-label follow-up phase, where restrictions on other MG-specific therapies, including rituximab, no longer exist.

To provide information on the possible effects of the study drug in the longer term, two follow-up return visits are included:

• Return visit after 36 weeks (+/- 21 days): checking for side effects, verifying current drugs and Prednisolone dose, QMG, MG-ADL, MG-QoL15, EQ5D and somatic status.

Note that QMG testing should be performed 12 hours after the last ingestion of cholinesterase inhibitors.

• Return visit after 48 weeks (+/- 21 days): checking for side effects, verifying current drugs and Prednisolone dose, QMG, MG-ADL, MG-QoL15, EQ5D and somatic status.

Note that QMG testing should be performed 12 hours after the last dose of cholinesterase inhibitors.

See Section 20, Appendix 1 for flow chart

8.7 Study end

The study ends when the last patient has completed the last follow-up.

The study may be terminated prematurely if it turns out that the treatment causes a large number of undesirable serious events or if patient recruitment cannot be fulfilled within a reasonable time limit.

Decisions on early termination of studies are made by the sponsor / coordinating investigator.

When the study is completed, the Ethical Review Board and the Medical Products Agency will be informed of this in accordance with set time limits.

9 Study participants

9.1 Inclusion criteria

1. Patients with oculobulbar, bulbar or generalized MG ≥ 18 years of age and ≤ 12 months after onset of generalized symptoms or neurophysiological detection of generalized disease.

2. The diagnosis of MG should be made by the following tests:
Clinical neurological status with motor symptoms compatible with MG and ≥ 2 of the following:

a Positive serological test for anti-acetylcholine receptor antibodies (AChR),

and/or

b. For MG typical findings on neuro-physiological testing of neuromuscular transmission by single fiber electromyography (SFEMG) and / or repetitive nerve stimulation (RNS),

and/or

c. Positive anticholinesterase test, e.g. edrophonium chloride test or improvement of MG symptoms with oral cholinesterase inhibitors at the discretion of the treating physician.

3. MGFA clinical classification class II to IV at screening.

4. Quantitative MG score ≥ 6 at screening

5. Women of childbearing potential must have a negative pregnancy test.

6. Patients must have given written informed consent.

7. Patients must be able and willing to follow all study procedures.

9.2 Exclusion criteria

1. Weakness affecting only ocular or periocular muscles (MGFA class I).

2. MG crisis at screening (MGFA class V).

3. Thymectomy already performed. To avoid difficulties in evaluating the effect of the study drug, thymectomy, in cases where it is deemed indicated, should be planned for the follow-up period, i.e. only after the first 24 weeks.

4. Strong suspicion of thymoma and where thymectomy according to the treating physician should be performed within 24 weeks.

5. Activecancer, unless adequately treated.

6. Pregnancy or breast-feeding.

7. Any ongoing acute or chronic viral or systemic bacterial infection including HIV, latent hepatitis B, which is clinically significant in the opinion of the study physician, and which has not been treated with appropriate antibiotic / antiviral drugs.

8. Severe heart failure (New York Heart Association Class IV) or severe uncontrolled heart disease

9. Previous use of immunosuppressive drugs including rituximab, azathioprine, ciclosporin and MMF. The use of Prednisolone at a dose of ≤40mg / d within 3 months and IVIG and PLEX 12 months from the screening date does not constitute an exclusion criterion. Please also note that this does not apply to treatment with immunosuppressive drugs / corticosteroids (excluding rituximab) for indications other than MG, provided that > 12 months have elapsed since the end of treatment.

10. Hypersensitivity to the active substance, to murine proteins or to any of the other excipients in the study medicinal product

11. Participation in any other clinical drug study or exposure to any other study drug, study product or study procedures within 30 days before screening.

12. Any medical condition which, in the opinion of the study physician, may interfere with the patient's participation in the study, poses any additional risk to the patient, or which complicates the assessment of the patients.

13. Vaccination within 4 weeks before inclusion.

9.3 Criteria for discontinuing participation

The patient can at any time during the course of the study interrupt his / her participation in the study without explaining the reason for this in more detail. The reason why the patient cancels his participation should, if possible, be registered. Data collected up to and including the cessation of study participation will be used in the final analysis of the study.
The examiner may also interrupt the patient's continued participation in the study;
- between the time of inclusion and the administration of study drugs if new data emerges that may pose risks during rituximab treatment, such as the detection of active hepatitis B infection, cancer or other factors that mean that inclusion and exclusion criteria are no longer met.
- after administration of study drugs: as the study involves only one administration of study drugs, continued participation will only be discontinued if the treating physician considers that continued participation in follow-up controls would in any way have negative consequences for the subject's mental or physical well-being.

Patients who have been included in the study but have not received study medication before discontinuation of participation, can be replaced with a new participant. Patients who have received study drugs are not substituted by additional participants.
The sponsor reserves the right to terminate the study prematurely based on scientific, administrative and / or ethical reasons. The study may be terminated prematurely if it turns out that the treatment causes a large number of undesirable serious events or if patient recruitment cannot be fulfilled within a reasonable time limit. Decisions on early termination are made by the sponsor / coordinating investigator.

9.4 Screening log

Patients evaluated for possible participation in the study are noted on a screening log at each center. Possible participants are only identified with initials, no personal data may be documented.

9.5 Study personell

The principal investigator at each center is responsible for conducting the study at the center. If study-specific tasks are delegated, it must be documented in writing to which persons and which study-specific tasks have been delegated.

10 Study drug

10.1 Study drug

Mabthera® 500 mg concentrate for infusion, solution
Active substance: Rituximab
ATC-kod: L01XC02

10.2 Description of study drug

Rituximab is a genetically engineered chimeric mouse / human monoclonal antibody consisting of a glycosylated immunoglobulin with human IgG1 constant regions and murine variable regions of the light and heavy chains. The antibody is produced by mammalian (Chinese hamster ovary) cell suspension culture and purified by affinity chromatography and ion exchange, as well as specific viral inactivation and purification steps.

According to the SmPC, Rituximab has been shown to have high specificity for the CD20 antigen on B cells. Toxicity studies in cynomolgus monkeys have not shown any other effects other than the expected pharmacological elimination of B cells in peripheral blood and lymphatic tissue.

Toxicity studies have been performed with cynomolgus monkeys at doses up to 100 mg / kg (treatment on gestational day 20-50) and showed no evidence of fetal toxicity caused by rituximab. In contrast, pharmacologically dose-dependent low levels of B cells in the lymphoid organs were observed in the fetus, which remained postnatally and was followed by a decrease in IgG levels in the newborn animals affected. The number of B cells returned to normal in these animals within 6 months after birth and did not affect the response to vaccination.

Standard tests to examine mutagenicity have not been performed as such tests are not relevant for this molecule. No long-term animal studies have been performed to determine the carcinogenicity of rituximab.

Specific studies to determine the effect of rituximab on fertility have not been performed. In general toxicity studies in cynomolgus monkeys, no adverse effects on male or female reproductive organs were observed.

10.3 Placebo

Sodium Chloride, solution for infusion, 9 mg/ml

Active substance: Sodium Chloride

ATC-kod: B05BB01

10.4 Packaging, labeling and handling of study drug

Study drugs (rituximab / placebo) are prepared and labeled at APL.

Mabthera (500 mg / 50 ml vial) is mixed in 250 ml NaCl infusion bags. 50 ml of NaCl is aspirated, 50 ml of Mabthera (500 mg) is added to a strength of 2 mg / ml. For placebo, use 250 ml NaCl in the corresponding infusion bags.

Study drugs are labeled with the date and time of preparation and the date and time of the latest time for use. The maximum shelf life of the study drug is 36 hours according to APL's guidelines.

The study drug is delivered in ready-made infusion bags to the centers during refrigerated transport and temperature log by World Courier. In most cases, delivery will take place within 24 hours.

10.5 Randomization

Study staff at the center contact APL for randomization or contact the study coordinator / study coordinating research nurse at Karolinska for help with randomization via APL.

10.6 Blinding and emergency code breeaking

The study is double-blind and labeling of study drugs (rituximab / placebo) is done by APL.

APL sends a code envelope for the patient in question in connection with sending the study drug to the center. The code envelopes must be stored in a safe place at the center and only opened when information about the given treatment (rituximab / placebo) is deemed necessary for the continued care of the patient. The integrity/status of envelopes will be checked at study center closure.

10.7 Permitted/Not permitted treatments in different study phases

Before screening

I] Permitted: Corticosteroids: Oral - Prednisolone ≤40mg / d within 3 months before screening.

IVIG: Within 12 months before screening

PLEX: Within 12 months before screening

Cholinesterase inhibitors: no restrictions on pre-screening use.

II] Not permitted: Immunosuppressants: Rituximab, azathioprine, cyclosporine, MMF, methotrexate or other immunosuppressants for the treatment of MG. Treatment (excluding rituximab) discontinued> 12 months ago for another indication is permitted

Methylprednisolone pulse

After screening, until randomization

I] Permitted: A stable dose of cholinesterase inhibitor and Prednisolone according to standard schedule if clinically indicated and PLEX and IVIG according to clinical routine.

II] Not permitted: Immunosuppressants: Rituximab, azathioprine, cyclosporine, MMF, methotrexate or other immunosuppressants.

Methylprednisolone pulse

After randomization, until week 8

I] Permitted: Prednisolone: For patients taking Prednisolone, the predetermined schedule of 8 weeks of tapering applies, i.e. 40mg / d, with a reduction of 5mg / v to discontinuation.

Extension of the tapering schedule in clinically clearly unstable patients, but with the aim of

reach a maximum dose of 10mg / day from week 9.

Cholinesterase inhibitors: No restrictions.

IVIG / PLEX: No restrictions.

II] Not permitted: Immunosuppressants: Rituximab, azathioprine, cyclosporine, MMF, methotrexate or other immune suppressants.

Methylprednisolone pulse

After randomization, week 9-24

I] Permitted: Prednisolone: Max dose 10mg / day

Cholinesterase inhibitors: No restrictions.

Immunosuppressants: azathioprine, cyclosporine, MMF or methotrexate may be initiated if clinically indicated;

Azathioprine: azathioprine, up to 2.5 mg / kg / day divided into one or two doses

Ciclosporin: up to 3mg / kg / day divided into two doses

Methotrexate: up to 20mg / week

II] Not permitted: Immunosuppressants: Rituximab or another immunosuppressant.

IVIG / PLEX

Methylprednisolone pulse

After randomization, week 25-48

I] Permitted: Prednisolone: No restrictions.

Methylprednisolone: No restrictions.

Cholinesterase inhibitors: No restrictions.

Immunosuppressants: No restrictions.

10.8 Clinical worsening, rescue treatments

In case of persistent or worsening MG symptoms, rescue treatment should be considered.

Primarily prolonging or reintroducing Prednisolone up to 40mg / d with a new phasing-out schedule; reduction 5mg / week to discontinuation (total treatment time 8 weeks)

Secondarily:

IVIg: total dose 1g / kg, spread over 2-3 days, alternatively

PLEX: A plasma volume is exchanged 3-5 times over 10-14 days

For more info about the above immunomodulatory therapies and the respective degree of evidence at MG, please see the National MG care program at www.snema.se (tab "Documents")

NOTE! Any newly initiated MG immunotherapy (excluding azathioprine, methotrexate and ciclosporin) or dose increase of Prednisolone> 10 mg / d during weeks 9-24 will by definition be considered rescue treatment. This also includes rituximab.

10.9 Study drug documentation, accountability

The investigator is responsible for the study drugs that are received, administered, or destroyed at the center being registered on the intended documents by delegated staff.

When administering study drugs, this is documented with information such as who gave the drug, patient, date, and time.

10.10 Treatment after study end

After the end of the study, the patient will be treated according to a clinical assessment by the treating physician.

11 Assessement of safety and efficacy

11.1 Blood samples

Blood samples for analysis of CBC including diff., Na, K, Creatinine, Asat, Alat, CRP, Glucose, hepatitis B screening and total IgG are taken at screening and analyzed at the local laboratory at each hospital.

Blood samples for analysis of drug antibodies and markers of disease activity, such as acetylcholine receptor antibodies, are taken in the study for evaluation of tertiary issue / exploratory analyzes in the study.

Analysis of autoantibodies is performed at Clinical Immunology / Transfusion Medicine, Karolinska University Laboratory after completion of the study.

Antibody antibodies and samples for exploratory analyzes will be sent to the Center for Molecular Medicine (CMM) at Karolinska Institutet (KI).

In total, a blood volume of about 50 ml is drawn from the patient during the study.

11.2 Quantitative Myastenia Gravis scale, QMG

The QMG scale is used in the study to evaluate primary, secondary, and tertiary study objectives.

The scale is used for quantitative evaluation of MG status and will be carried out by testers who have not participated in the administration of the study drug. The scale consists of 13 domains that are scored 0-3 as well as information about MGFA and subjective health status.

Evaluations on the scale take about 20-30 minutes to complete and are carried out during visits to the center.

11.3 Myasthenia gravis activities of daily life scale, MG-ADL

The MG-ADL scale is used in the study to evaluate secondary and tertiary study objectives.

The scale consists of eight items such as speech, swallowing, double vision, and mobility where the patient himself estimates activities in daily life on a three-point scale.

Filling in the form takes about 5 -10 minutes and is carried out by the patient during a visit to the center together with the research nurse.

11.4 EuroQol 5-dimensions quality of life, EQ5D

The EQ5D health survey is used in the study to evaluate tertiary study objectives.

EQ5D is an instrument for measuring health and has been developed by EuroQol Group. The form contains a part with 5 items where the patient classifies his health, such as mobility, main activities, pain / discomfort, and a part with a scale where the patient estimates his current state of health based on the criteria best to worst imaginable.

Filling in the form takes about 5 -10 minutes and is carried out by the patient during a visit to the center together with the research nurse.

11.5 Quality of life in myasthenia gravis, MG-QOL15

MG-QOL15 is used in the study for evaluation of secondary and tertiary study objectives.

MG-QOL15 is a short form for evaluation for certain aspects of quality of life associated with myasthenia gravis.

The form consists of 15 items, such as frustration over the condition, difficulty eating, difficulty speaking, difficulty walking, etc. The patient himself estimates his function on a five-point scale based on criteria from not at all to very much based on how the statements are true during the last 4 weeks.

Filling in the form takes about 5 -10 minutes and is carried out by the patient during a visit to the center together with the research nurse.

11.6 Neurophysiology

Repetitive nerve stimulation (RNS) is performed at baseline and after 24 weeks for centers where neurophysiology is located locally.

Surface electrodes are applied to a muscle, the muscle nerve is stimulated with a series of electric shocks at rest, after maximum activation of the muscle, 1 min and 3 min after activation and the amplitude is measured.

RNS is performed in the proximal arm muscle (deltoid) and facial muscle (nasalis) and measures disturbed neuromuscular transmission by measuring decrements.

The patient should not take cholinesterase inhibitors within 12 hours before the examination.

- RNS procedure:
  - o Low-frequency nerve stimulation (3 Hz) 10 times at rest
  - o Muscle activation 20 seconds and then again 3 Hz stimulation 10 times
  - o 1 minute after activation again low-frequency nerve stimulation (3 Hz) 10 times
  - o 3 minutes after activation again low-frequency nerve stimulation (3 Hz) 10 times
- Measurement parameters:
  - o% amplitude decrement (reduction) between 1st and 4th muscle response (compound motor action potential; CMAP)
  - o% area decrement between 1st and 4th CMAP
  - o CMAP amplitude in absolute numbers at rest (mV)

The examination is performed at a neurophysiological lab at each hospital. Patients admitted to centers that do not have a neurophysiological lab in the hospital do not need to do this test.

11.7 Adverse Event, AE and Serious Adverse Event, SAE.

Follow-up regarding AE and SAE will be carried out during the telephone follow-ups and all physical visits that the patient carries out at the center.

The patient is informed at the start of the study to contact the center if new or worsening symptoms should arise. If symptoms worsen significantly, or if the patient experiences an MG crisis (difficulty breathing), the patient should inform the physician and make an evaluation visit as soon as possible or no later than 48 hours after the onset of symptoms. Treatment of the patient then takes place in accordance with standard care for the patient's clinic or hospital.

12 Management of adverse events

As a reference safety information for assessing whether an adverse event is expected or not, Mabthera SmPC / SmPC will be used.

12.1 Definitions

12.1.1 Adverse events (AE)

Any adverse medical event or deterioration of an existing medical condition, whether related to treatment or not.

12.1.2 Serious adverse events (SAE)

Any accidental medical event occurring at any dose:

- results in death
- is life threatening
- causes hospital stay or extended hospital stay (see also section 12.5 regarding hospitalization for MG relapses)
- results in permanent or temporary disability
- results in a congenital injury / malformation
- a medically important event that involves danger to the patient or that could have resulted in any of the above if not taken care of

12.1.3 Suspected, unexpected, serious adverse events (SUSAR)

A reaction / event that is unexpected, serious, suspected to be caused by the treatment and that has not been described before.

12.2 Assessement of adverse events

12.2.1 Assessement of seriousness

Any adverse medical event should be classified by the examiner as mild, moderate, or severe.

Mild: The event does not affect the person's normal life.

Moderate: The event causes deterioration of function but does not affect health. The event causes discomfort and / or discomfort / obstacles.

Severe: The event causes impairment of function or ability to work or poses a health risk to the person.

12.2.2 Assessement of causality

Related / probably related: Clinical event, including abnormal laboratory analyzes, which occur within a reasonable time after administration of the intervention / study product. It is unlikely that the event can be attributed to the underlying disease or other drugs.

Possibly related: Clinical event, including laboratory analyzes, occurring within a reasonable time after administration of the intervention / study product. The event can also be explained by the underlying disease or other drugs.

Not related: Clinical event, including abnormal laboratory analyzes, which may be temporarily related to the administration of the intervention / study product. The event is unlikely to be related to the intervention / study product and may better be explained by other drugs or underlying disease.

12.3 Methods for detection of adverse events

At the start of the study, the patient is informed to contact the center in the event of new or worsening symptoms. During telephone follow-ups and return visits that the patient carries out at the clinic, the patient will also be asked if any unwanted medical events have occurred since the last time.

12.4 Reporting of adverse events

12.4.1 Reporting of adverse events (AE)

All adverse medical events are noted in a special AE form, where the severity is assessed as severe or non-serious.

12.4.2 Reporting of serious adverse events (SAE)

Serious adverse events should be reported to the sponsor on a special SAE form within 24 hours of the study center becoming aware of the SAE.

Follow-up information describing the outcome and management of SAE should be reported as soon as that information is available. The original report must be inserted in the investigator binder.

12.4.3 Reporting of suspected, unexpected, serious adverse events (SUSAR)

The sponsor is responsible for all relevant information about suspicious, unforeseen, serious adverse reactions being registered and reported to the EudraVigilance database.

SUSARs that are fatal or life-threatening are reported to the EudraVigilance database as soon as possible and no later than 7 days after the occurrence has become known to the sponsor. Relevant follow-up information must then be submitted within a further 8 days. Other SUSARs are reported as soon as possible and no later than within 15 days after they have come to the sponsor's notice.

Information about SUSAR that occurs during the study will be compiled by the sponsor and sent out to the principal examiner at all participating centers quarterly in the form of a CIOMS form.

12.5 Follow up of adverse events

Adverse Event, AE

AE that occurs after the study drug has been administered until the visit week 48 will be documented, summarized, and analyzed in the study. If treatment is needed, this is done in accordance with the protocol and based on the medical assessment of the treating physician.

AE that is judged to be caused by the underlying disease or previously known diseases will not be registered as AE in the study. Should the underlying disease worsen during the study, this may be considered an AE.

AEs will be followed up until they are “resolved” or until the patient's participation in the study is completed.

Serious Adverse Event, SAE

SAE that arises from the start of administration of study drugs until the patient ends his / her participation in the study will be documented and reported, as well as summarized and analyzed after the study.

Planned hospitalization / surgery for illness or condition that the patient had before the start of the study will not be registered as SAE.

Note that hospitalization that is primarily caused by exacerbated MG symptoms (MG crisis) should not be considered SAE. If the deterioration and hospitalization are secondary to, for example, pneumonia, the event must be reported as SAE.

SAEs will be followed up until they are “resolved” or until the patient's participation in the study is completed.

12.6 Annual safety reporting (Development Safety Update Report, DSUR)

During the study, an annual safety report (DSUR) will be sent to the Medical Products Agency and the Regional Ethics Review Board.

The report contains a summary of the SAEs and SUSARs that have occurred, a summary assessment of the safety of the patients included in the study and information on the benefit-risk assessment has changed since the study was approved.

13 Statistics, data handling and archiving

13.1 Deciding on number of study subjects

Based on results reported in Sanders DB et al.[2], our own experience, and a shorter phasing out of cortisone than used in most protocols, it is estimated that ≤40% in the placebo group achieve the primary endpoint of a daily Prednisolone dose ≤10mg with QMG ≤ 4. With 25 patients in each arm, the power will be 0.85 with an alpha = 0.05 for a two-sided test of 80% reaching the primary endpoint of the active arm.

[Note, due to slow recruitment the funder of the study, the Swedish MRC, requested a revised power calculation. March 15th 2018 the following text was submitted, which was approved May 25th 2018:
"The study regards a study drug being administered on only one occasion, with determination of main endpoints at 4 and 6 months, respectively. Possible drop-outs are therefore limited to study participants actively interrupting the study, including the follow-up visits that the protocol stipulates, or that dies before these visits. Of a total of 23 included study participants, none have interrupted. One study participant died due to a medical condition not considered directly related to the study medication. A revised power calculation with the same input variables (without loss) shows that with the same number of patents in each treatment arm: 20 per arm (N = 40), power = 81%; 22.5 per arm (N = 45), power = 85%; 25 per arm (N = 50), power = 89%. Based on interim results, it is therefore reasonable to expect a dropout rate <5%. In order to maintain a power of at least 80% and a certain margin for any minor imbalance in the randomization, n≥45 is needed." The power calculation provided in the paper is based on this revised calculation.]

13.2 Analysis plan

Differences in the primary endpoint will be tested on an intention-to-treat basis using Fisher's exact test. As a secondary analysis, the primary endpoint will also be analyzed with log-binomial regression, adjusted for any observed baseline differences that have occurred despite randomization.

Patients who have received disease-modulating MG treatment as indicated in section 10.7 as "Not allowed" after randomization and before the assessment visit at 16 weeks will be counted as non-responders.

In addition to the primary efficacy variable, a few secondary outcome measures of particular clinical relevance have been selected to reduce the risk of false-positive findings. Differences in the change of these continuous measures will be tested with the Mann-Whitney U-test. P-values will be adjusted with Bonferroni correction for these three tests. To estimate 95% confidence intervals around the difference, linear regression with robust standard errors will be used, without adjustment for the number of tests. Patients who have received disease-modulating MG therapy as indicated in section 10.7 as "Not allowed" after randomization and before the assessment visits at 16 and 24 weeks will be censored from the time these treatments were administered.

Other measures are tertiary or exploratory.

A challenge in clinical trials is the management of dropouts, which risks introducing a selection error if response data are only available to the patients who remain in the study. As this study only involves study drug administration at baseline, we expect that the drop-out rate, and thus the need for imputation for those who have left the study, will be limited. If the dropout rate is higher than 10%, imputation will be performed, partly with multiple imputation with explicit modeling of the treatment outcome as a function of baseline variables (age, gender, disease activity), partly through so-called "last observation carried forward", where values for patients who left the study are replaced with their most recently observed value. However, it is very important to emphasize the importance of including complete study data on as many subjects as possible.

13.3 Data handling and Case Report Forms, CRF

Data collected in the study are registered by the study staff in paper CRF (source document), where certain information is also entered in specially modified pages in the Swedish MG register (MGreg). This concerns rating scales, dates of follow-up visits, cortisone dose, possible side effects and any need for other MG-specific therapy (rescue treatments). This part of MGreg belongs to the decision support and not the quality register part of MGreg. MGreg is located on the same platform as the other sub-registries in the Swedish Neuroregister and is accessed in the usual way by logging in from a computer with an Internet connection. For patients who give consent to registration in the MGreg quality registry part, results from the assessment scales are also transferred to this part. However, the study does not require the consent of the quality registry section.

Patient data is collected according to the protocol and registered continuously in paper CRF (source documents), with duplication of certain information in MGreg CRF. For each contact occasion, there is a page with the necessary

information to fill in. When extracting data from MGreg (the decision support part), the patient's name and social security number are deleted, so that only the patient number in MGreg remains. This number is thus the link to the person's name and social security number and is available only to the investigators and nurses who work locally at the patient's clinic and people who work with quality assurance of the study. All withdrawals from the registry are logged.

Information entered in MGreg is locally available to each investigator even after the end of the study.

All blood samples leaving the hospital will also be marked with this patient number only. No unauthorized person will have access to the code key.

Collected data originates in source documents at the clinic such as lab reports and other test results and results.

13.4 Data processing

Data processing and statistical analysis will be carried out by the coordinating investigator in collaboration with biostatisticians at the Department of Medicine, Karolinska University Hospital Solna. A compilation of study data is distributed to participating investigators when the study is completed.

Data processing can begin after the last patient has completed the last visit in the study.

13.5 Archiving

Data collected during the study will be archived for at least 10 years after the study is completed.

14 Quality control

14.1 Source data

Source data in the study are the patient's medical record, paper CRF for all contact sessions, questionnaires / assessment scales as well as transcripts such as analysis answers and lab lists.

At each center, a source data document will be drawn up specifying what is the source data for the center.

14.2 Monitoring

To ensure that the study is conducted according to the protocol, that data is collected, documented, and reported in accordance with ICH-GCP (Good Clinical Practice) and current ethical and regulatory requirements, the study will be monitored by an external party before the study begins, during the study and after that the study has been completed.

The monitoring also aims to ensure that the subject's rights, safety, and well-being are met and that the data in the CRF are filled in, correct and in accordance with the source data.

14.3 Access to source data

Study monitors can gain access to medical records and source data after a confidentiality agreement has been signed by the medical record manager at the clinic and monitor. The accuracy of data entered in MGreg will be checked against source documents.

15 Ethics

15.1 Ethical review board and Medical Products Agency

The sponsor / coordinating investigator is responsible for ensuring that the application for approval for the implementation of the study is obtained from the Regional Ethics Review Board and the Medical Products Agency.

Additions to or significant changes to the protocol can be made after the application for an addition / amendment has been approved by the Regional Ethics Review Board and / or the Medical Products Agency.

15.2 Ethical conditions for the study

The study will be conducted in accordance with the protocol and the latest version of the Declaration of Helsinki, ICH-GCP and other applicable ethical and regulatory laws, rules, and requirements.

Patients are informed orally and in writing about the study and are informed that participation in the study is completely voluntary and that participation can be interrupted at any time, regardless of the reason, without this affecting the patient's future treatment in any way.

The patient is informed that the data collected until the interruption will be analyzed.

Data collected during the study are coded so that no individual can be distinguished.

15.3 Benefit risk assessement

During the study, blood samples will be taken and an intravenous catheter inserted for the administration of study drugs. For most patients, the placement of intravenous catheters and needle punctures for blood sampling is well tolerated. In rare situations, however, they can cause bleeding, bruising, swelling, coagulation in the vein, leakage of medication or solution into the surrounding tissues, and possibly infection at the site of insertion of the needle or catheter. Sampling and insertion of an intravenous catheter will be performed according to clinical practice by staff with extensive experience in this field.

As with all medicines, rituximab can cause side effects, although well tolerated compared to other biological medicines. The side effects of rituximab are usually mild or moderate. The investigator will discuss possible side effects with the patient and explain the risks and benefits of rituximab before treatment. Because rituximab is being studied in MG patients, not all possible side effects are known.

Very common side effects (affects at least 1 in 10 patients) include fever, itching, redness of the skin, nausea, headache.

Common side effects (affects 1 or more in 100 patients and less than 10 in 100 patients) include: reduced platelet count, abdominal pain, constipation, vomiting, diarrhea, stomach discomfort after meals, nausea, chest discomfort, chills, infusion-related reaction, anaphylactic reaction, edema, fever, tiredness, weakness, cold sores, cold, viral infection, stomach flu, bronchitis, severe infection, muscle aches, back and neck pain, pain in limbs and joints, muscle cramps, dizziness, disturbances in taste, tingling of the body, vertigo, high blood pressure, difficulty or pain when urinating, spontaneous erection, upper respiratory tract infection, pneumonia, urinary tract infection, joint infection, cough, irritation or pain in the throat, nasal congestion, itchy skin, rash, hair loss, and dry skin .

In addition, serious infections, sepsis, and infusion reactions have been reported in patients receiving rituximab and these are identified safety hazards.

Patients receiving rituximab will have a weakened immune system, which may affect the patient's ability to fight viral and bacterial infections. Patients treated with rituximab have an increased risk of worsening certain chronic infections, such as hepatitis B.

Based on what has been described above, the risks that study participation entails are considered acceptable given that the study population includes patients with new-onset moderate to severe MG (moderate to severe effects on ADL), which is a condition that risks deteriorating further if immunomodulatory treatment is not instituted, in turn involving other types of risks such as osteoporosis, poor blood sugar control and other side effects of cortisone.

The patient's condition may improve, remain the same, or worsen during the study. If the study and possibly other studies with rituximab in MG are positive, this may lead to benefit for other patients, as clinical evidence for efficacy with this drug is then substantiated. It is worth mentioning that today there is a complete lack of approved treatments for MG and that clinical practice is based entirely on proven experience. We believe that this will affect national practice for the treatment of MG.

15.4 Procedure for collection of informed consent

The responsible investigator or other delegated co- investigator first informs the patient orally about the study structure, purpose, examinations, risks and discomforts, as well as alternative treatments and provides the written patient information and consent form to the patient. The information is provided during inpatient stay or during an outpatient visit at the investigator's clinic. The patient is informed that participation in the study is completely voluntary and that he / she can interrupt his / her participation in the study at any time and without specifying the reason. The patient is informed that data collected until interruption will be analyzed in the study.

The responsible investigator can delegate to the study nurse to provide parts of the oral information on their own, such as how the administration of study drugs is done and routine for telephone follow-ups.

The patient is given the opportunity to read the patient information in peace and quiet and think through their decision well before deciding. If the patient, after reading the information, is interested in participating in the study, he / she is given the opportunity to meet the examiner and is then given the opportunity to ask further questions and have them answered.

Consent to participation in the study, after information and time to reflection, is given by the patient personally providing written consent to participate. The investigator who provided information about the study also signs and dates the consent form. The signed original of the consent form is kept at each clinic and the patient receives a copy of patient information including the consent form. The investigator documents in the patient's medical record that the person agrees to participate in the study. No examinations within the framework of the study may be carried out before the patient has given his or her written consent.

16 Insurance

During the study, the study participants are covered by the regular insurance covering medical care in Sweden.

17 Funding

The study is funded by the Swedish Research Council via a framework grant for clinical treatment research

18 Publication of results

The results from the study will be compiled in a summary clinical study report within one year after the end of the study. The report is distributed in accordance with current European guidelines.

The study results will be published in scientific journals and / or presented at international meetings and conferences.

19 References

1. Fang F, Sveinsson O, Thormar G, Granqvist M, Askling J, Lundberg IE, et al.
   The autoimmune spectrum of myasthenia gravis: a Swedish population-based study.
   J Intern Med. 2014.

2. Sanders DB, Hart IK, Mantegazza R, Shukla SS, Siddiqi ZA, De Baets MH, et al.
   An international, phase III, randomized trial of mycophenolate mofetil in myasthenia gravis.
   Neurology. 2008;71(6):400-6.

3. Bedlack RS, Simel DL, Bosworth H, Samsa G, Tucker-Lipscomb B, Sanders DB.
   Quantitative myasthenia gravis score: assessment of responsiveness and longitudinal validity.
   Neurology. 2005;64(11):1968-70.

4. Group MS.
   A trial of mycophenolate mofetil with prednisone as initial immunotherapy in myasthenia gravis.
   Neurology. 2008;71(6):394-9.

5. Wolfe GI, Herbelin L, Nations SP, Foster B, Bryan WW, Barohn RJ.
   Myasthenia gravis activities of daily living profile.
   Neurology. 1999;52(7):1487-9.

6. Muppidi S, Wolfe GI, Conaway M, Burns TM, Mg C, Mg-Qol15 Study G.
   MG-ADL: still a relevant outcome measure.
   Muscle Nerve. 2011;44(5):727-31.

87

20 Annex 1, Study flow chart

	**Screening**	**Baseline** (week 0)	**Week 2** (+/- 2 days) Phone	**Week 4** (+/- 2 days) Visit	**Week 8** (+/- 4 days) Phone	**Week 12** (+/- 4 days) Phone	**Week 16** (+/- 21 days) Visit	**Week 20** (+/- 21 days) Phone	**Week 24** (+/- 21 days) Visit	**Week 36** (+/- 21 days) Visit	**Week 48** (+/- 21 days) Visit
Informed consent	X										
Inclusion-/exclusion criteria	X	X									
MGFA (class II-IV)	X	X									
Medical history	20.1.1.1.1.1.1.1										
Physical exam	X[1]	X		X			X		X	X	X
Concomitant medication[2]	20.1.1.1.1.1.1.2	X	X	X	X	X	X	X	X	X	X
Blood tests	20.1.1.1.1.1.1.3 [3]	X[8]					X[11]		X[11]		
Pregnancy test[4]	20.1.1.1.1.1.1.4										
QMG	X	X		X			X		X	X	X
Repetitive neurophysiology, RNS [5]	X								X[11]		
Randomisation	X										
EQ5D[6]	X	X		X			X		X	X	X
MG-QoL15[6]	X	X		X			X		X	X	X
MG-ADL[7]	X	X		X			X		X	X	X
Study drug[8,9, 10]		X									
Prednisolone dose		X	X	X	X	X	X	X	X	X	X
AE/SAE (continuous)		X	X	X	X	X	X	X	X	X	X

[1] Previous and current diseases, concomitant medications, blood pressure, heart rate, body temperature, age, sex, height, weight and disease activity. [2]To be updated at all visits. [3] Haematology including differential count, electrolytes, creatinine, liver enzymes C-reactive protein, glucose, hepatitis B screening, total immunoglobulin G levels. [4] Fertile women. [5] Optional. No cholinesterase inhibitors within 12 hours before examination. [6] Myasthenia Gravis-Quality of Life. [7] Myasthenia Gravis-Activities of Daily Living. [8] Serum sample to be collected before administration of study drug.
[9] 1 000 mg paracetamol, 50 mg prednisolone and 10 mg Cetirizine ≥ 45 min before administration of study drug. [10] Heart rate, blood pressure and body temperature to be recorded before and after administration of study drug.[11] At or close to visit

21 Annex 2, MGFA classification

MGFA clinical classification

Class I: Any ocular muscle weakness; may have weakness of eye closure. Strength of all the other muscles is normal.

Class II: Mild weakness affecting muscles other than ocular muscles; may also have ocular muscle weakness of any severity.

IIa. Predominantly affecting limb, axial muscles, or both. May also have lesser involvement of oropharyngeal muscles.

IIb. Predominantly affecting oropharyngeal, respiratory muscles, or both. May also have lesser or equal involvement of limb, axial muscles, or both.

Class III: Moderate weakness affecting muscles other than ocular muscles; may also have ocular muscle weakness of any severity.

IIIa. Predominantly affecting limb, axial muscles, or both. May also have lesser involvement of oropharyngeal muscles.

IIIb. Predominantly affecting oropharyngeal, respiratory muscles, or both. May also have lesser or equal involvement of limb, axial muscles, or both.

Class IV: Severe weakness affecting muscles other than ocular muscles; may also have ocular muscle weakness of any severity.

IVa. Predominantly affecting limb, axial muscles, or both. May also have lesser involvement of oropharyngeal muscles.

IVb. Predominantly affecting oropharyngeal, respiratory muscles, or both. May also have lesser or equal involvement of limb, axial muscles, or both.

Class V: Defined as intubation, with or without mechanical ventilation, except when employed during routine postoperative management. The use of a feeding tube without intubation places the patient in class IVb.

22 Annex 3, Study drug infusion/observation

Mabthera/Placebo infusion observation

Date: ______________________

Pre-medication 45-60 min before start of infusion:

T. Deltison 50 mg, 1 st
T. Alvedon 500 mg, 2 st } **Admin (time):** _________
T. Cetirizin 10 mg, 1 st

Subject no: _______________________

Initials: ________________

Study physician: ____________________

	Time:	Temp:	Blood pressure:	Pulse:	Notes/Signature
**Before start:**					

Start of infusion. Time:	End of infusion. Time:	Time:	Infusion-speed:	Blood pressure:	Pulse:	Notes/Signature<br>(infusion reactions)

	Time:	Temp:	Blood pressure:	Pulse:	Notes/Signature
**After infusion:**					

First infusion (”slow drip rate”)	
**0-30 min:**	**50 ml/hr (100 mg/hr)**
**30-60 min:**	**100 ml/hr (200mg/hr)**
**60-90 min:**	**150 ml/hr (300mg/hr)**
**90 min and after:**	**200 ml/hr (400 mg/hr)**

Blood pressure is checked before the start of the infusion and after the end of the infusion.

Observe the patient during the ongoing infusion and check blood pressure and reduce the infusion rate if the patient experiences side effects.

23 Annex 4, QMG

RINOMAX

MGFA och Kvantitativt MG status

Återbesöksdatum: Tid:

Site-nummer:________________

Patientnummer:______________

Initialer:________

	0-6 tim	7-11 tim	12-24 tim	>24
Timmar sedan senaste kolinesterashämmare:	☐	☐	☐	☐

MGFA:
- ☐ 1 Symptom endast från ögon
- ☐ 2a Mild svaghet i extremitets-/axial muskulatur
- ☐ 2b Även eller enbart mild svaghet i bulbär muskulatur
- ☐ 3a Måttlig svaghet i extremitets-/axial muskulatur
- ☐ 3b Även eller enbart måttlig svaghet i bulbär muskulatur
- ☐ 4a Uttalad svaghet i extremitets-/axial muskulatur
- ☐ 4b Även eller enbart uttalad svaghet i bulbär muskulatur
- ☐ 5 Intuberad och/eller mekanisk ventilation

Cirkla korrekt ruta och skriv uppmätt värde i rutan

Kvantitativt MG status (13 domäner poängsätts 0-3)	0p	1p	2p	3p
1. Dubbelseende vid blick uppåt	120s	91-119s	11-90s	<11s
2. Ptos vid blick uppåt	120s	91-119s	11-90s	<11s
3. Grimasera	20ggr	11-19ggr	5-11ggr	<5ggr
(alt ansiktsmuskulatur): Sluter ögonlock	Normalt	Reducerad kraft	Ej mot motstånd	Kan ej sluta
4. Tuggning	20ggr	11-19ggr	5-11ggr	<5ggr
(alt sväljfunktion vatten)	Normalt	Minimal hosta	Kraftig hosta	Kan ej svälja
5. Räkning till 100 - Dysartri	100	41-100	5-40	<5
6. Armlyft höger	40ggr	25-39ggr	10-24ggr	<10ggr
(alt Framsträckta armar höger)	180s	60-179s	10-59s	<10s
7. Armlyft vänster	40ggr	25-39ggr	10-24ggr	<10ggr
(alt Framsträckta armar vänster)	180s	60-179s	10-59s	<10s
8. PEF % normalt för ålder, kön (se bild baksida)	76-140%	51-75%	26-50%	<26%
9. Fingerextension höger	70ggr	40-69ggr	10-39ggr	<10ggr
10. Fingerextension vänster	70ggr	40-69ggr	10-39ggr	<10ggr
11. Huvudlyft	30ggr	15-29ggr	5-14ggr	<5ggr
12. Benlyft höger dynamiskt	35ggr	21-34ggr	10-20ggr	<10ggr
(alt Benlyft höger statiskt 45 grader)	60s	40-59s	15-39s	<15s
13. Benlyft vänster dynamiskt	35ggr	21-34ggr	10-20ggr	<10ggr
(alt. Benlyft vänster statiskt 45 grader)	60s	40-59s	15-39s	<15s

Ev. Kommentarer:

Undersökare: Daterad: version 3 2016-12-12

Somatiskt status: ua ☐

Anmärkningar: ____________________

____

Vikt: __________ Längd: __________ Puls: __________ Blodtryck: __________

Oförändrad medicinering: ja ☐

ev. Dosjusteringar: ____________________

____

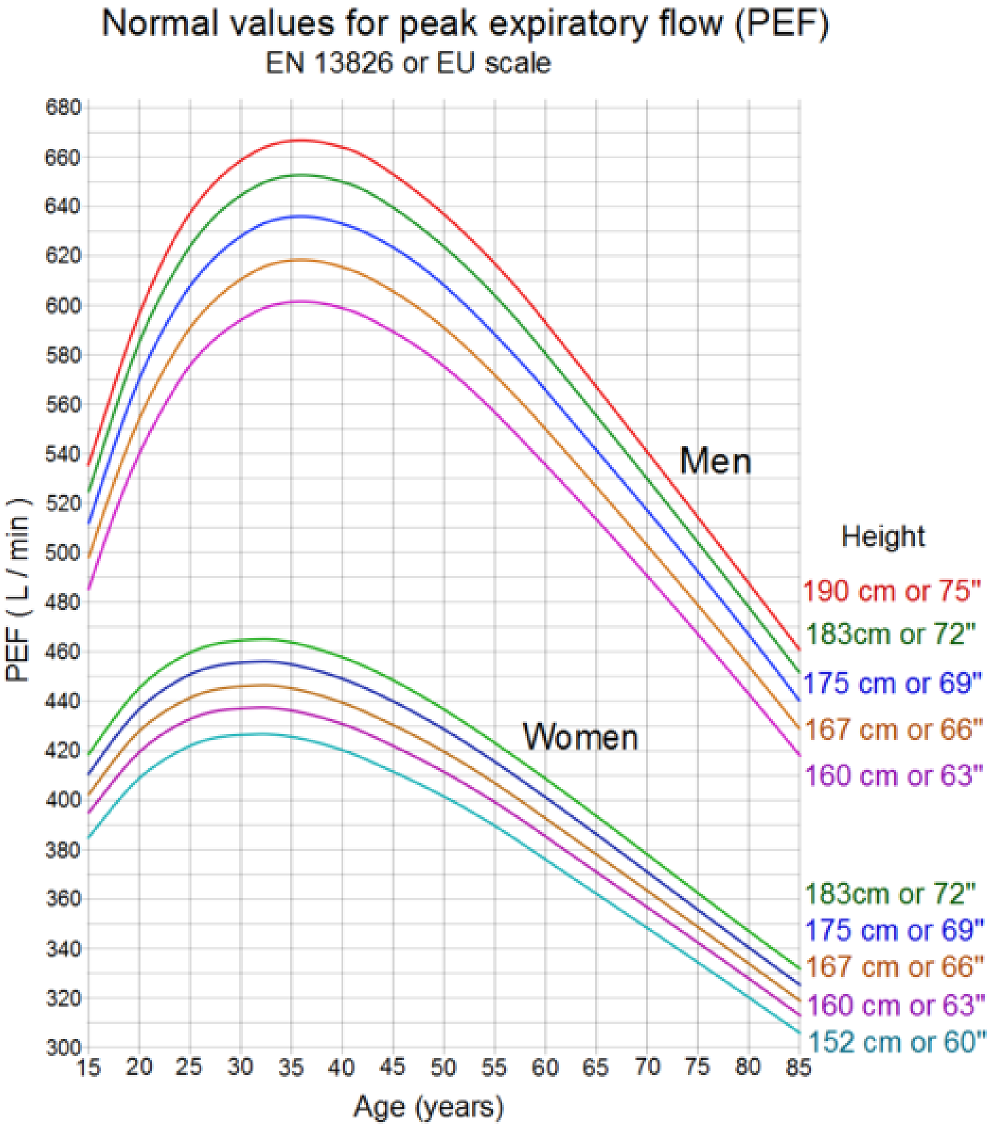

1041

24 Annex 5, MG-QoL15

Site Number:	Trial Visit:
Subject Number:	Date: ____/____/____ dd mm yyyy

Ange hur väl varje påstående stämde för dig (under de senaste 4 veckorna).

	Inte alls	Lite	Något	Ganska mycket	Väldigt mycket
	0	1	2	3	4
1. Jag är frustrerad över mitt tillstånd					
2. Jag har svårigheter med att använda ögonen					
3. Jag har svårigheter med att äta					
4. Jag har begränsat mina sociala aktiviteter på grund av mitt tillstånd					
5. Mitt tillstånd begränsar min förmåga att finna nöje i fritidsintressen och roliga aktiviteter					
6. Jag har svårigheter med att uppfylla min familjs behov					
7. Jag måste planera med hänsyn till mitt tillstånd					
8. Min yrkesskicklighet och min arbetssituation har påverkats negativt					
9. Jag har svårigheter att tala					
10. Jag har svårigheter med att köra bil					
11. Jag är deprimerad över mitt tillstånd					
12. Jag har svårigheter med att gå					
13. Jag har svårigheter med att ta mig runt på allmänna platser					
14. Jag känner mig överväldigad av mitt tillstånd					
15. Jag har svårigheter med att sköta mina kroppsvårdsbehov					

Livskvalitet vid myastenia gravis
"MG-QOL15"
Burns TM, et al., *Muscle and Nerve* 2008

Summa MG-QOL15-poäng

25 Annex 6, MG-ADL

Site Number:	Trial Visit:
Subject Number:	Date: ____/____/____ dd mm yyyy

MG-skala över aktiviteter i dagliga livet

Poster	Grad 0	Grad 1	Grad 2	Grad 3	Poäng (0,1,2,3)
1. Tal	Normalt	Intermittent sluddrigt eller nasalt tal	Konstant sluddrigt eller nasalt tal, men kan förstås	Svårighet att förstå tal	
2. Tuggning	Normal	Tröttsamt med fast föda	Tröttsamt med mjuk föda	Ventrikelsond	
3. Sväljning	Normal	Sällan förekommande kvävnings-händelse	Ofta förekommande kvävnings-händelse som kräver ändrad kost	Ventrikelsond	
4. Andning	Normal	Andfåddhet vid ansträngning	Andfåddhet vid vila	Respirator-beroende	
5. Nedsatt förmåga att borsta tänder eller kamma håret	Ingen	Extra ansträngning, men inget behov av viloperioder	Behov av viloperioder	Kan inte göra någon av dessa funktioner	
6. Nedsatt förmåga att resa sig från en stol	Ingen	Lätt, använder ibland armarna	Måttlig, använder alltid armarna	Uttalad, behöver hjälp	
7. Dubbelseende	Inget	Förekommer, men inte dagligen	Dagligen, men inte konstant	Konstant	
8. Hängande ögonlock	Inget	Förekommer, men inte dagligen	Dagligen, men inte konstant	Konstant	

Total ADL-poäng vid MG (posterna 1-8) ____________

Clinical Evaluator's Signature:	Date: ____/____/____ dd mm yyyy

MG-ADL v13/May/15 ECU-MG-302 Sweden (Swedish)

26 Annex 7, EQ5D

Site Number:	Trial Visit:
Subject Number:	Date: ___/___/___ dd mm yyyy

EQ-5D-3L

Hälsoenkät

Svensk version för Sverige

(Swedish version for Sweden)

It is recommended QOL be completed by the subject prior to any other visit procedures.

Subject Signature:	Date: ___/___/___ dd mm yyyy

Sweden (Swedish) © 1990 EuroQol Group. EQ-5D™ is a trade mark of the EuroQol Group

EQ-5D-3L _ ECU-MG-302 _ Sweden (Swedish)

Site Number:	Trial Visit:
Subject Number:	Date: __/__/____ dd mm yyyy

Markera, genom att kryssa i en ruta i varje nedanstående grupp (så här ☑), vilket påstående som bäst beskriver Ditt hälsotillstånd i dag.

Rörlighet

Jag går utan svårigheter ☐

Jag kan gå men med viss svårighet ☐

Jag är sängliggande ☐

Hygien

Jag behöver ingen hjälp med min dagliga hygien, mat eller påklädning ☐

Jag har vissa problem att tvätta eller klä mig själv ☐

Jag kan inte tvätta eller klä mig själv ☐

Huvudsakliga aktiviteter *(t ex arbete, studier, hushållssysslor, familje- och fritidsaktiviteter)*

Jag klarar av mina huvudsakliga aktiviteter ☐

Jag har vissa problem med att klara av mina huvudsakliga aktiviteter ☐

Jag klarar inte av mina huvudsakliga aktiviteter ☐

Smärtor/besvär

Jag har varken smärtor eller besvär ☐

Jag har måttliga smärtor eller besvär ☐

Jag har svåra smärtor eller besvär ☐

Oro/nedstämdhet

Jag är inte orolig eller nedstämd ☐

Jag är orolig eller nedstämd i viss utsträckning ☐

Jag är i högsta grad orolig eller nedstämd ☐

It is recommended QOL be completed by the subject prior to any other visit procedures.

Subject Signature:	Date: __/__/____ dd mm yyyy

2

Sweden (Swedish) © 1990 EuroQol Group. EQ-5D™ is a trade mark of the EuroQol Group

EQ-5D-3L _ ECU-MG-302 _ Sweden (Swedish)

Site Number:	Trial Visit:
Subject Number:	Date: ____/____/____ dd mm yyyy

Till hjälp för att avgöra hur bra eller dåligt ett hälsotillstånd är, finns den termometer-liknande skalan till höger. På denna har Ditt bästa tänkbara hälsotillstånd markerats med 100 och Ditt sämsta tänkbara hälsotillstånd med 0.

Vi vill att Du på denna skala markerar hur bra eller dåligt Ditt hälsotillstånd är, som Du själv bedömer det. Gör detta genom att dra en linje från nedanstående ruta till den punkt på skalan som markerar hur bra eller dåligt Ditt nuvarande hälsotillstånd är.

Ditt nuvarande hälsotillstånd

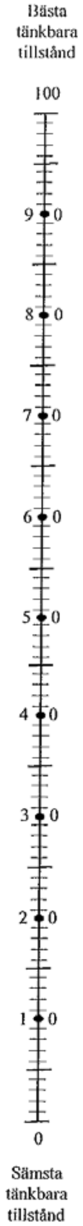

It is recommended QOL be completed by the subject prior to any other visit procedures.

Subject Signature:	Date: ____/____/____ dd mm yyyy

Sweden (Swedish) © 1990 EuroQol Group. EQ-5D™ is a trade mark of the EuroQol Group

EQ-5D-3L _ ECU-MG-302 _ Sweden (Swedish)

1059
