# Supplementary material for: Efficacy and Safety of Rituximab for New-Onset Generalized Myasthenia Gravis: The RINOMAX Randomized Clinical Trial
Source: JAMA Neurol. 2022 Sep 19;79(11):1105–12. doi: 10.1001/jamaneurol.2022.2887 (PMC9486640; doi:10.1001/jamaneurol.2022.2887)
Supplement: Supplement 2. — eFigure 1. Change in disease activity score from randomization, with censoring at time of rescue treatment eFigure 2. Change in disease activity score from randomization eFigure 3. Acetylcholine receptor antibody concentrations, change from baseline to week eTable. Stratification for daily Prednisolone doses [file jamaneurol-e222887-s002.pdf]

Supplemental Online Content

Piehl F, Eriksson-Dufva A, Budzianowska A, et al. Efficacy and safety of rituximab for new-onset myasthenia gravis: the RINOMAX randomized clinical trial. *JAMA Neurol*. Published online September 19, 2022. doi:10.1001/jamaneurol.2022.2887

eFigure 1. Change in disease activity score from randomization, with censoring at time of rescue treatment

eFigure 2. Change in disease activity score from randomization

eFigure 3. Acetylcholine receptor antibody concentrations, change from baseline to week

eTable. Stratification for daily Prednisolone doses

This supplemental material has been provided by the authors to give readers additional information about their work.

© 2022 Piehl F et al. *JAMA Neurology.*

eFigure 1. Change in disease activity score from randomization, with censoring at time of rescue treatment

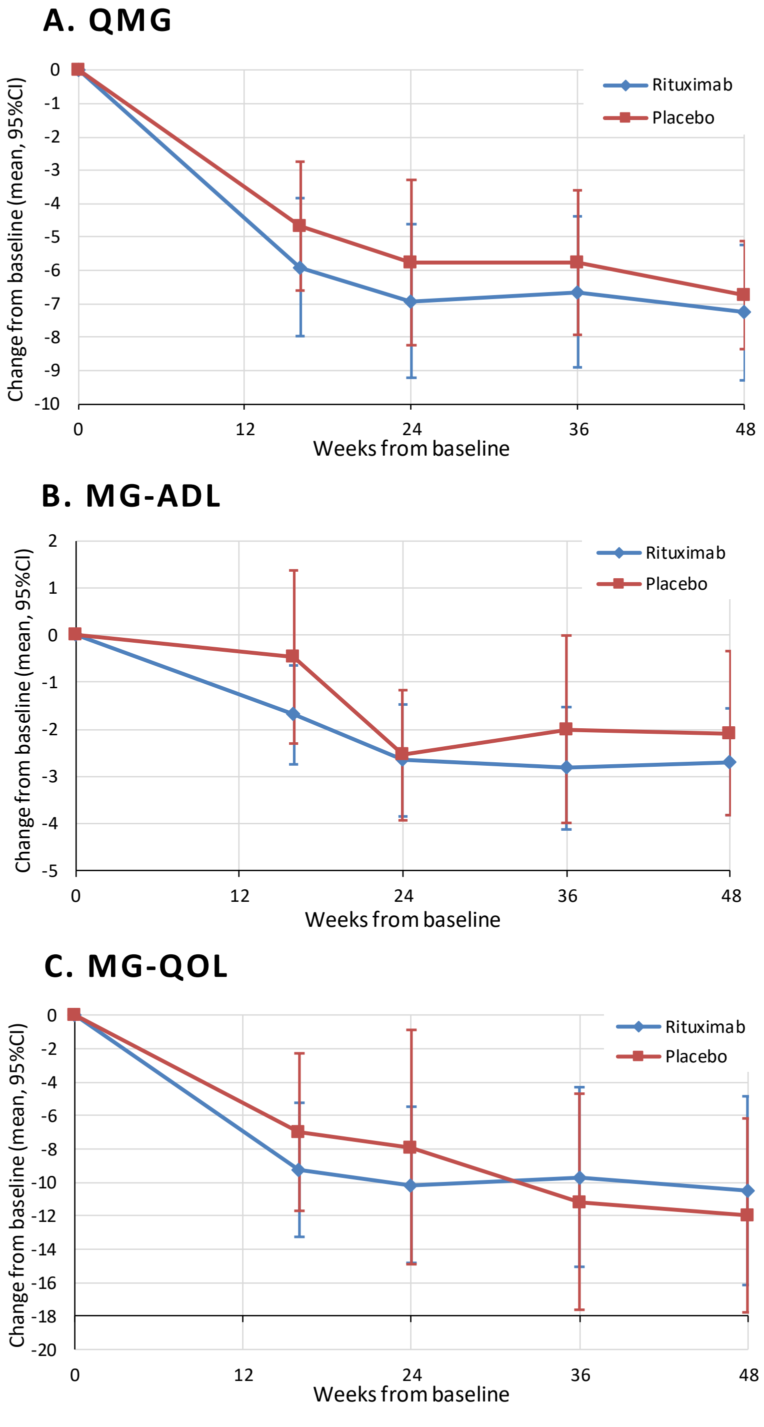

QMG, Quantitative Myasthenia Gravis score; MG-ADL, Myasthenia Gravis Activities of Daily Living score; MG-QoL, Myasthenia Gravis Quality of Life questionnaire score; 95% Confidence intervals estimated with robust (Huber-White) standard errors. Subjects were censored at time of rescue treatment, disproportionately reducing the placebo group over time (see Figure 2B in main manuscript).

© 2022 Piehl F et al. *JAMA Neurology.*

eFigure 2. Change in disease activity score from randomization

A. QMG

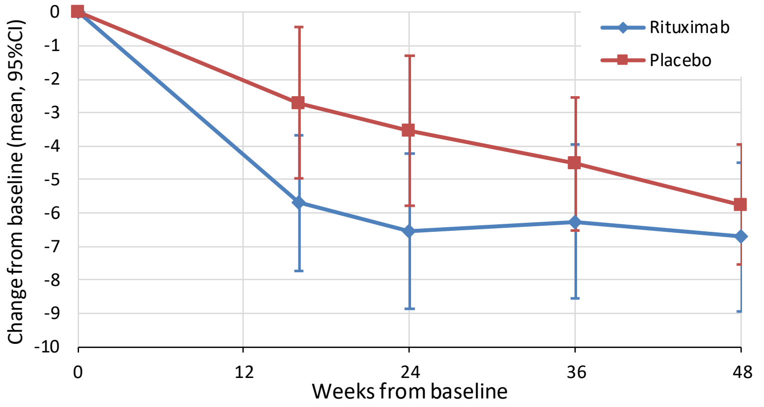

B. MG-ADL

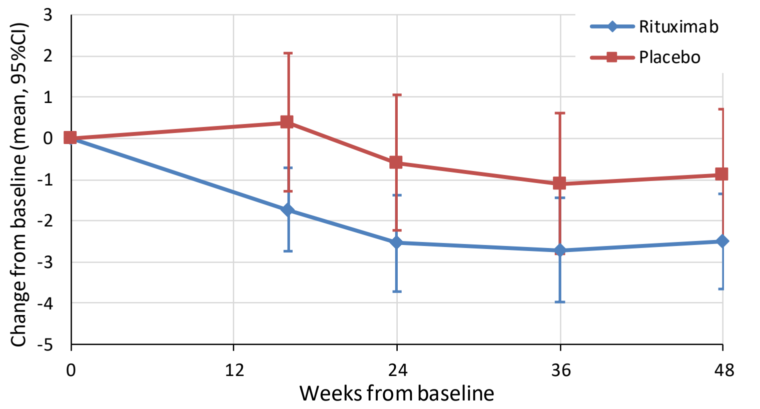

C. MG-QOL

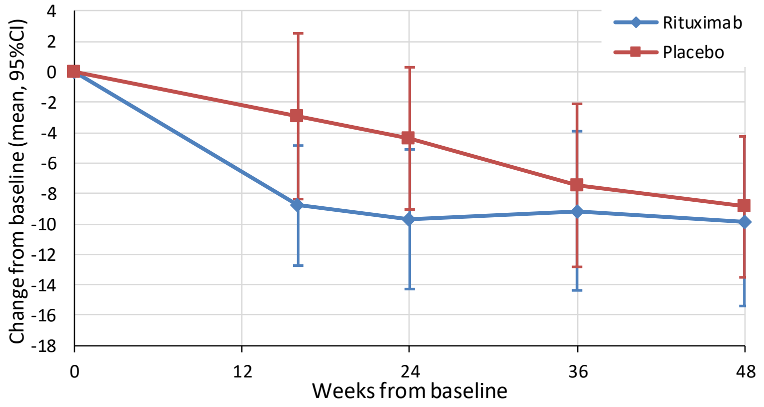

QMG, Quantitative Myasthenia Gravis score; MG-ADL, Myasthenia Gravis Activities of Daily Living score; MG-QoL, Myasthenia Gravis Quality of Life questionnaire score; 95% Confidence intervals estimated with robust (Huber-White) standard errors.

© 2022 Piehl F et al. *JAMA Neurology.*

eFigure 3. Acetylcholine receptor antibody concentrations, change from baseline to week 24

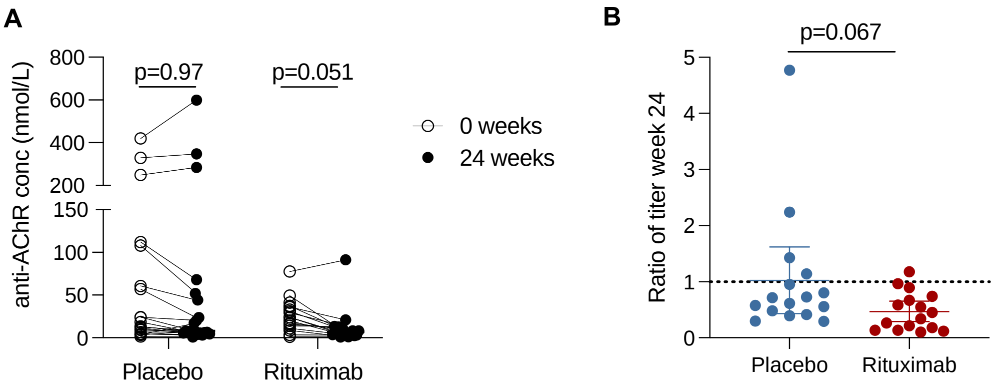

Figure legend:

(A) Change in acetylcholine receptor antibody concentrations from baseline to week 24.

(B) Ratio of acetylcholine receptor antibody concentrations at week 24 in relation to levels at baseline, i.e. concentration at week 24 divided by concentration at baseline.

© 2022 Piehl F et al. *JAMA Neurology.*

eTable. Stratification for daily Prednisolone doses

Daily prednisolone dose at baseline			
Group (freq, %)	≤10 mg	11-20 mg	>20 mg
Placebo	16 (73)	1 (4)	5 (23)
Rtx	13 (52)	5 (20)	7 (28)

Maximal daily prednisolone dose week 1 to 8			
Group (freq, %)	≤10	11-20	>20
Placebo	5 (23)	4 (18)	13 (59)
Rtx	7 (28)	7 (28)	11 (44)

Daily prednisolone dose at week 16			
Group (freq, %)	≤10	11-20	>20
Placebo	21 (95)	0 (0)	1 (5)
Rtx	24 (100)	0 (0)	0 (0)

© 2022 Piehl F et al. *JAMA Neurology.*
