# Supplementary material for: Efficacy and Safety of Rituximab for New-Onset Generalized Myasthenia Gravis: The RINOMAX Randomized Clinical Trial
Source: JAMA Neurol. 2022 Sep 19;79(11):1105–12. doi: 10.1001/jamaneurol.2022.2887 (PMC9486640; doi:10.1001/jamaneurol.2022.2887)
Supplement: Supplement 3. — Data sharing statement [file jamaneurol-e222887-s003.pdf]

Data Sharing Statement

Piehl. Efficacy and Safety of Rituximab for New-Onset Myasthenia Gravis. *JAMA Neurol*. Published September 19, 2022. doi:10.1001/jamaneurol.2022.2887

Data
Data available: Yes
Data types: Deidentified participant data, Data dictionary
Additional Information: Aggregated data
How to access data: fredrik.piehl@ki.se
When available: Requests for sharing of deidentified data will be considered upon reasonable request and in accordance with current legislation regarding protection of personal data. Additional group level data has been deposited with ClinicalTrials.gov and the EU clinical trials register.

Supporting Documents
Document types: None

Additional Information
Who can access the data: Researchers whose proposed use of the data has been approved
Types of analyses: Deidentified individual, meta-analysis
Mechanisms of data availability: With a signed data access agreement, with Investigators support
